# Experimental and computational studies of an antiplasmodial derivative of allantoin; antimycobacterial essential oil from *Cordia batesii* WERNHAM (Boraginaceae)

**DOI:** 10.1186/s13065-021-00742-5

**Published:** 2021-03-05

**Authors:** Eric Robert Tiam, Dominique Serge Ngono Bikobo, Ibrahim Mbouombouo Ndassa, Norbert Mbabi Nyemeck II, Auguste Abouem A Zintchem, Lawrence Ayong, Patrick Hervé Betote Diboué, Bruno Lenta Ndjakou, Joséphine Ngo Mbing, Dieudonné Emmanuel Pegnyemb

**Affiliations:** 1 Department of Organic Chemistry, Faculty of Science, University of Yaoundé I, P.O Box 812, Yaounde, Cameroon; 2 Department of Inorganic Chemistry, Faculty of Science, University of Yaoundé I, P.O Box 812, Yaounde, Cameroon; 3 Department of Chemistry, Higher Training College, University of Yaoundé I, P.O Box 47, Yaounde, Cameroon; 4 Centre Pasteur du Cameroun, Yaounde, Cameroon; 5 Centre for Research on Medicinal Plants and Traditional Medicine, Institute of Medical Research and Medicinal Plants Studies, Yaounde, Cameroon

**Keywords:** *Cordia batesii*, Allantoin, Batesiin, Spectroscopy, Essential oil, DFT, Molecular orbitals, *Plasmodium falciparum*, *Mycobacterium tuberculosis*

## Abstract

**Background:**

Chemical and pharmacological investigations were performed on the stems of *Cordia batesii* (Boraginaeae); chemical studies included quantum calculations applied on a newly described compound.

**Results:**

A new derivative of allantoin (**1**) named batesiin (**2**) was characterized. Thirteen other known compounds involving allantoin (**1**) were either isolated or identified. GC–MS enabled the identification of six compounds from a fraction containing essential oil. MeOH extract and some isolated compounds were tested in vitro against *Pf*7G8 CQS and *Pf* Dd2 CQR strains of *Plasmodium falciparum*; extract disclosed a moderate antiplasmodial activity (IC_50_ = 50 μg mL^−1^). Meantime, the CH_2_Cl_2_ extract and essential oil fraction were tested on a resistant mycobacterial strain of *Mycobacterium tuberculosis*; a potent antimycobacterial activity with a MIC = 9.52 μg mL^−1^ was deduced from essential oil. Density functional theory (DFT) calculations were carried on batesiin (**2**). Calculated chemical shifts at B3LYP/6-31G(d,p) and MPW1PW91/6-31G+(d,p) showed much better correlations with the experimental data. Time dependent DFT at B3LYP/6-31G+(d,p) displayed a major absorption band 3.01 nm higher than the experimental value.

**Conclusion:**

*Cordia batesii* can be considered as promising in search of compounds with antimalarial and antitubercular properties. DFT studies are very helpful when trying to learn more about the spectroscopic insights of a derivative of allantoin (**1**).

## Introduction

One of the main goals of World Health Organization (WHO) is to end the epidemics of neglected tropical diseases, tuberculosis (TB) and malaria (which remains the major public health and mortality problem in the tropics) by 2030 [1, 2]. In 2018, TB infected about 10.0 million people, mainly in WHO regions of South-East Asia (44%), Africa (24%) and the Western Pacific (18%); in parallel, about 213 million cases of malaria were found in the WHO African region. In the same year, half a million newly rifampicin-resistant TB cases were estimated. In general, 3.4% of new TB cases and 18% of formerly cured patients displayed either multidrug resistant TB or rifampicin-resistant TB (MDR/RR-TB) [3–5]. Trying to overcome the high cost or the shortage of drugs for treatment of malaria remains a challenge for chemists, and some authors proposed the synthesis of compounds disclosing an imidazole unit with efficient activities against malaria [6]. Recently, Al-Otaibi et al. [7] reported quantum calculations achieved on such derivatives; in their work, the authors evaluated the structural and electronic traits of those derivatives. ^1^H- and ^13^C-NMR spectroscopy is a central tool in the structure elucidation of organic compounds. A review by Lodewyk et al. [8] emphasized on computational predictions of NMR data on synthetic organic compounds and natural products. Natural products can be isolated from plants, which are considered to be an important source of major compounds in drug development because of their successful use in treating various human ailments since millenniums. In this context, searching for new natural products from medicinal plants could provide new ways for antimalarial and antitubercular drugs. Among these plants, some species of the genus *Cordia* (Boraginaceae) are reported to be useful in the treatment of tuberculosis, bronchitis and malaria [9].

The genus *Cordia* (Boraginaceae) is composed of trees or shrubs and is widespread in Central and South America, India, Asia and Africa [10]. Previous phytochemical investigations of plants from this genus reported the isolation and characterization of different classes of secondary metabolites including naphthoquinones, hydroquinones [11] or polyphenols [12]. Concurrently and based on some pharmacological surveys, essential oils from *C*. *curassivica* and *C*. *gilletii* appeared as active against some microbial strains [13, 14]. Biological activities and in silico investigations of *C*. *dichotoma* were recently reported [15]; the plant is also known to contain, apart from allantoin (**1**) [16, 17] which has been the subject of many quantum calculations [18, 19], fatty acids (FA) [20]. FA have been recognized as energy sources for *M*. *tuberculosis* inside host tissues and are supposed to induce dormancy in *Mycobacterium* bacilli [21, 22]. A few *Mycobacterium* bacilli were inhibited by *C*. *sebestena* extracts as results of biological analyses [23]. Sebestenoid D is a component of the latter species and was a matter of density functional theory (DFT) studies, with its optimized molecular geometry and HOMO–LUMO plot as outcomes [24, 25]. Highest occupied molecular orbital (HOMO) and lowest unoccupied molecular orbital (LUMO) were also calculated during a survey aiming to check the coherence between experimental and theoretical NMR and IR data; this investigation was done on a compound with an imidazole unit [26] like allantoin (**1**). Other simulations completed on a particular range of complex natural products involved IR as well as electronic transitions for UV analyses through time dependent DFT (TD-DFT); findings were afterwards compared to experimental results [27].

Despite the intensive work performed on some *Cordia* species, no or less investigation has been done on *Cordia batesii* species, maybe because of its limited geographic location. In our continuing search of secondary metabolites with powerful antiplasmodial and antitubercular activities, chemical investigations were carried on the stems of *Cordia batesii*, a forest shrub growing in the central and western regions of Cameroon. This paper describes the isolation of a new derivative of **1** named batesiin (**2**) along with other compounds. In vitro activities regarding extracts of stems and some isolated compounds against two CQR strains of *Plasmodium falciparum* were examined, when essential oil from the plant was tested against a resistant mycobacterial strain of *Mycobacterium tuberculosis*. The detailed characterization of **2** was investigated based on experimental NMR and UV–visible spectroscopic analyses; DFT at B3LYP/6-31G(d,p) [28, 29] and MPW1PW91/6-31G+(d,p) [30, 31] and TD-DFT simulations at B3LYP/6-31G+(d,p) were then undertaken on the alleged structure. These quantum calculations were applied on **2** for two main reasons: the compound is described for the first time, its NMR data are closed to chemical shifts of **1** but the UV–visible spectra of both seem different. DFT calculations at B3LYP/6-311G++(d,p) were also performed to check some electronic and thermodynamic properties of **2**.

## Results

### Experimental results

**Fig. 1:**
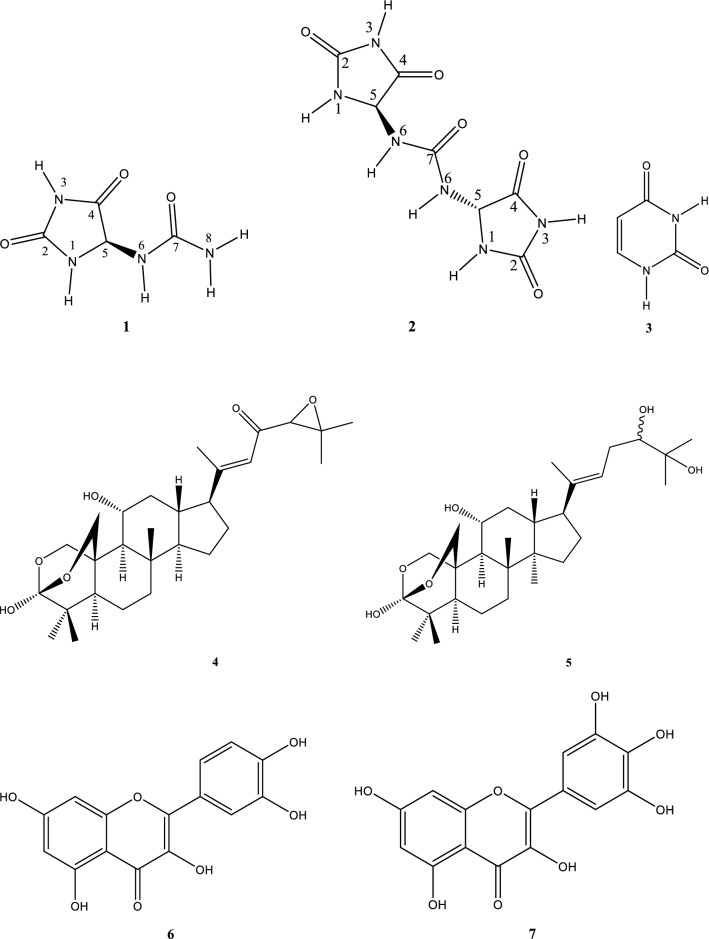

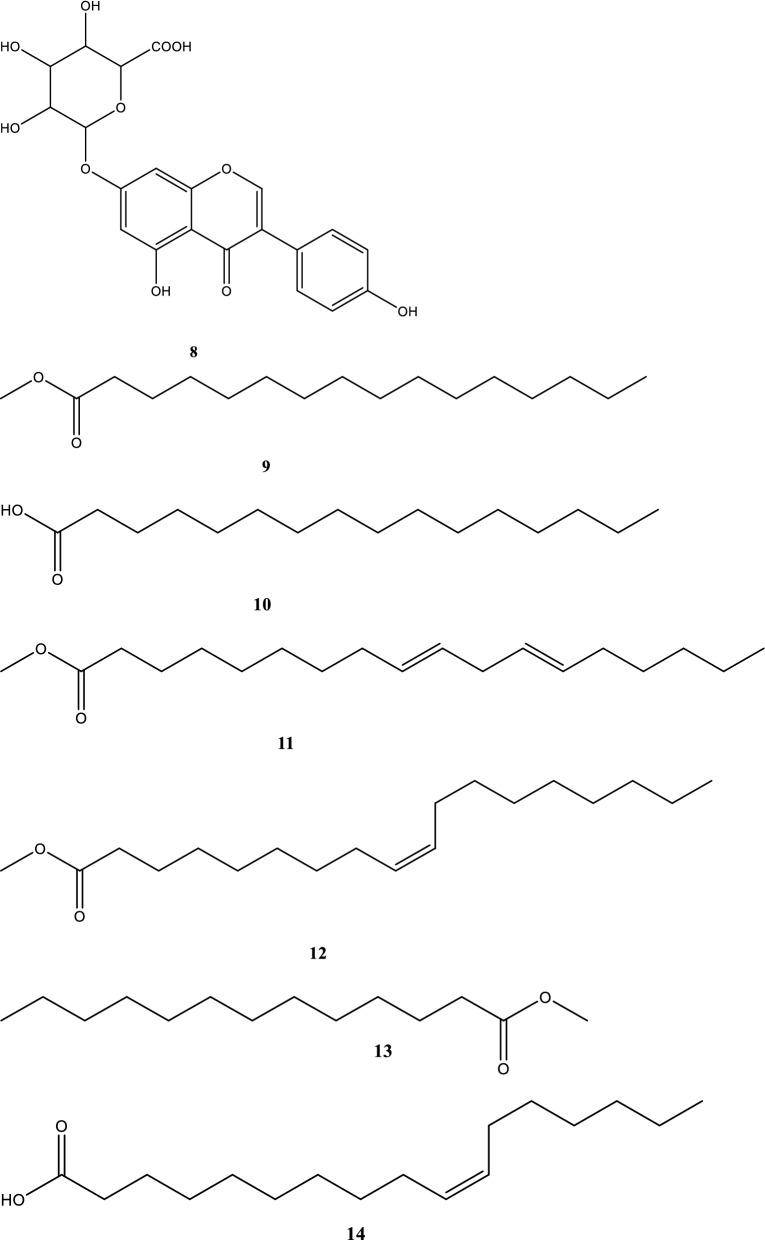
Structures of the isolated/identified compounds (**1–14**) from the stems of *C*. *batesii*

Compounds **1**, **3**–**14** were identified based on comparison of their physical and spectral data with authentic samples or those already reported (Fig. 1). Their assignments were consistent with structures of: allantoin (**1**) [32], pyrimidine 2,4-(1*H*,3*H*)-dione (**3**) [33], cordialin A (**4**) and cordialin B (**5**) [34], quercetin (**6**) [35], myricetin (**7**) [36], genistein 4′-*O*-glucuronide (**8**) [37], methyl palmitate (**9**) [38], palmitic acid (**10**) [39], methyl (9*E*,12*E*)-octadeca-9,12-dienoate (**11**) [40], methyl oleate (**12**) [41], methyl tridecanoate (**13**) [42] and (*Z*)-octadec-11-enoic acid (**14**) [43].

### Characterization of compound 2

Compound **2** was isolated as a white solid from CH_2_Cl_2_/MeOH mixture. Its molecular formula was deduced as C_7_H_8_N_6_O_5_ from the ESI-MS signal at *m/z* 257.4 [M+H]^+^ and from the HR-LC/MS signal at *m/z* 279.1603 [M+Na]^+^, in accordance with seven degrees of unsaturations. Additional data from the ESI-MS spectrum displayed other peaks at *m/z* 159.4 [M_1_+H]^+^ and 181.6 [M_1_ + Na]^+^ which are characteristics of allantoin (**1**) [44]. These preliminary data suggested a close relationship between allantoin (**1**) and compound **2**. The ^1^H-NMR spectrum of **2** exhibited a very prominent peak at δ_H_ 5.30 (2H, d*, *^*3*^* J*(H,H) = 2.0 Hz, H–C(5)) ppm. Moreover, we observed from the same spectrum some signals attributable to protons attached to heteroatoms (especially to nitrogen) with chemical shifts at δ_H_ 8.04 [2H, s, H–N(1)], 10.51 [2H, s, H–N(3)], 6.86 [2H, d, ^*3*^* J*(H,H) = 2.0 Hz, H–N(6)] ppm (Table 1).

**Table 1:** ^1^H- and ^13^C-NMR 1D and 2D spectroscopic data of **1** and **2** (500 and 125 MHz in DMSO-*d*_6_) in ppm

Position	Allantoin (1) [50, 61]		Batesiin (2)	
	δ_C_	δ_H_	δ_C_	δ_H_ (m, *J*)
2	156.7	–	156.8	–
4	173.4	–	173.6	–
5	62.3	5.23	62.4	5.30 (d, 2.0 Hz, 2H)
7	157.4	–	157.4	–
H–N(1)	–	8.04	–	8.04 (s, 2H)
H–N(3)	–	10.62	–	10.51 (s, 2H)
H–N(6)	–	6.88	–	6.88 (d, 2.0 Hz, 2H)

The ^13^C-NMR spectrum of **2** showed four remarkable signals at 157.4 [C(2), 2C], 173.6 [C(4), 2C], 62.4 [C(5), 2C] and 156.8 [C(7), 1C] ppm; when considering that **2** contains seven carbon atoms, the number of aforementioned signals presume the occurrence of a symmetry. The DEPT 135 NMR spectrum of **2** revealed one signal at δ_C_ 62.4 [C(5)] ppm indicating one methine group. These observations were confirmed by its HSQC spectrum which indicates a correlation between the proton at δ_H_ 5.30 ppm [H–C(5)] and the said carbon. Two remaining signals from the ^13^C NMR spectrum are observable at δ_C_ 48.6 and 54.9 ppm and are suggestive of signals of MeOH and CH_2_Cl_2_ respectively; this assertion is strengthened by correlations between signals at δ_C_ 48.6 and δ_H_ 3.17 ppm in one side, and signals at δ_C_ 54.9 and δ_H_ 5.74 ppm from the same HSQC spectrum [45].

**Fig. 2:**
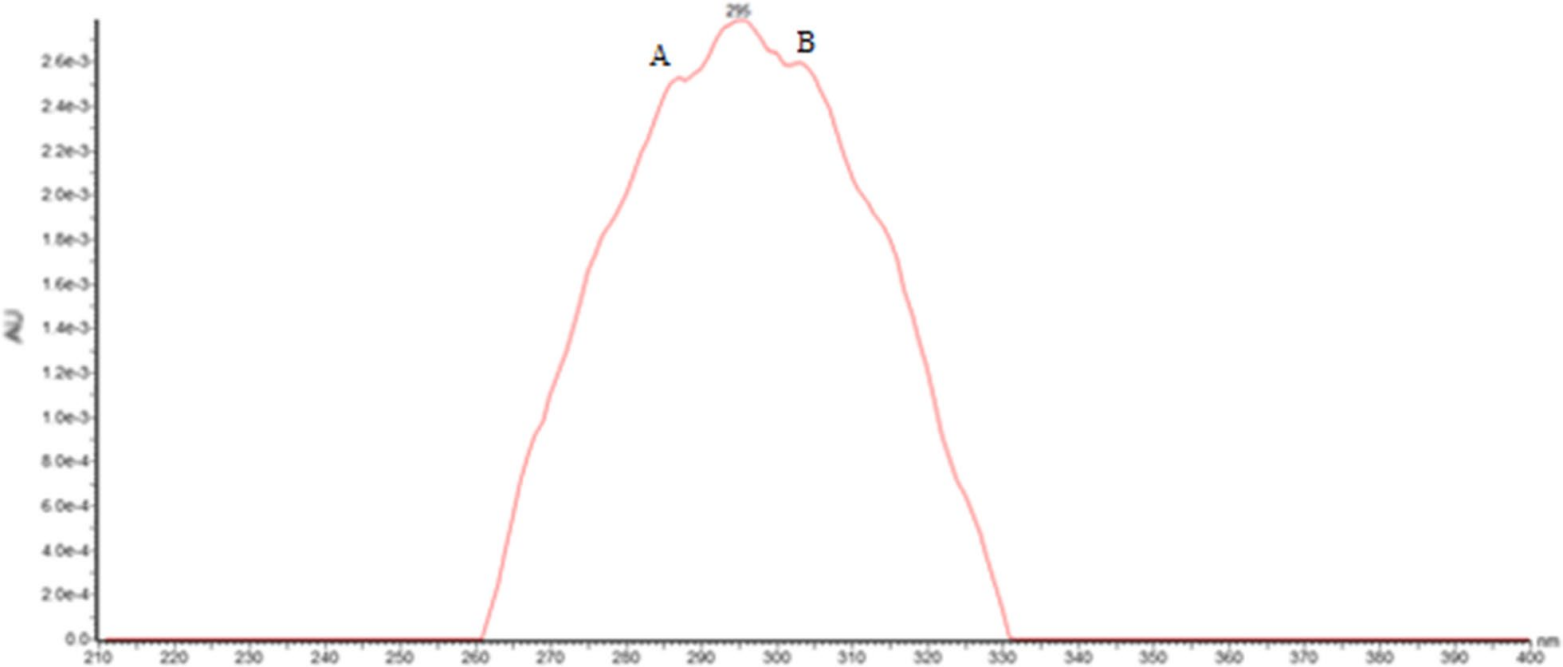
UV–visible spectrum of batesiin (**2**)

The HMBC spectrum exhibited noticeable correlations between protons at δ_H_ 5.30 ppm [H–C(5)] and the carbon atoms at δ_C_ 173.6 ppm [C(4)] and 156.8 ppm [C(7)], between protons at δ_H_ 6.88 ppm [H–N(6)] and carbon atoms at δ_C_ 173.6 [C(4)], 62.4 [C(5)] and 156.8 [C(7)]. Other correlations were found between nitrogenous protons at δ_H_ 8.04 [H–N(1)] ppm and carbon atoms at δ_C_ 157.3 ppm [C(2)], δ_C_ 173.6 ppm [C(4)] and 62.4 ppm [C(5)]. The UV spectrum of **2** (Fig. 2) exhibited one major maximum at λ_max_ = 296 nm, different from values of **1** [19].

Lakshmanan et al. [44] confirmed through an X-ray analysis that the occurring enantiomer of **1** is its (*S*) one. A thorough analysis of all the spectra and comparison with data from the literature revealed that compound **2** is described for the first time as a new derivative of allantoin (**1**); it was identified as (*S*,*S*)-1,3-bis(2,5-dioxoimidazolidin-4-yl)urea, trivially named batesiin (**2**). Table 1 shows some NMR data of allantoin (**1**) and batesiin (**2**); it strengthens the agreement of a close relationship between those two compounds in terms of NMR spectroscopic data.

### Biological properties

**Fig. 3:**
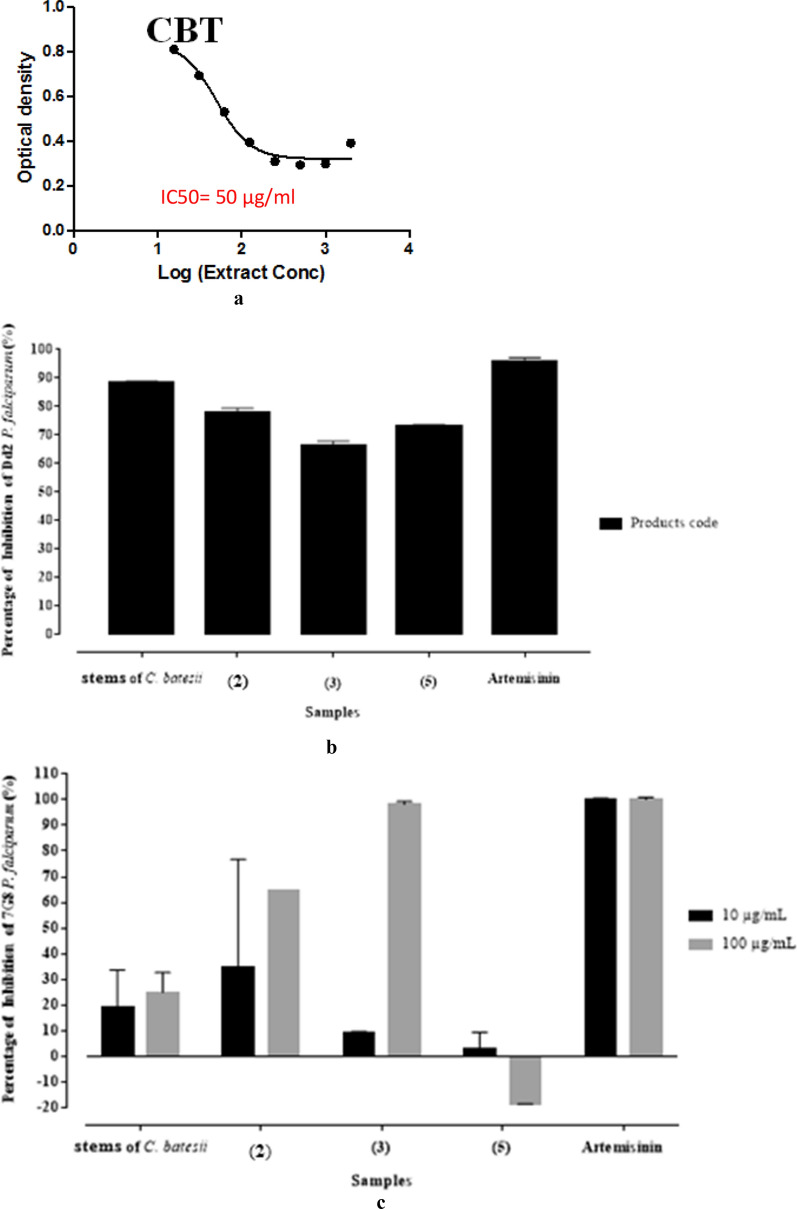
Result of twofold dose response analysis of the extract of *C*. *batesii* against Dd2 *P*. *falciparum* strain (**a**). Inhibition percentage of stems of *C*. *batesii* and isolated compounds against *P. falciparum* Dd2 strain (**b**). Inhibition percentage of stems of *C*. *batesii* and isolated compounds against *P. falciparum* 7G8 strain (**c**)

The antimalarial efficiency of screening against *P. falciparum* Dd2 and 7G8 (CQR) strains of the MeOH extract of stems of *C*. *batesii* and compounds **2**, **3**, **5** and **6** was performed according to the Sybr Green I fluorescence-based assays [46]. The results are presented in Fig. 3; they indicate the IC_50_ of extract of stems of *C*. *batesii* and the percentage of growth inhibition against Dd2 and 7G8 *P. falciparum* strains respectively.

Figure 3a mentioned that the MeOH extract of stems discloses an IC_50_ = 50 μg mL^−1^ against Dd2 *P*. *falciparum* strain which can be considered as a moderate activity. It appears from Fig. 3b that, apart from artemisinin (95.75% of inhibition) used as reference, the MeOH extract has the highest antiplasmodial activity with 88.24% percentage inhibition followed by **2** with approximately 78% of growth inhibition against Dd2 strain. Compounds **3** and **5** showed high activity (> 65%) of inhibition, exhibiting a growth inhibition of Dd2 strain *P. falciparum* with percentages corresponding to 66.43% and 72.99% respectively.

When tested against 7G8 *P. falciparum* strain, MeOH extract and compounds **2**, **3** and **5** displayed the percentages of growth inhibition of 18.82%, 34.26%, 10.04% and 2.84% at 10 μg mL^−1^ respectively. The same extract and isolated compounds at 100 μg mL^−1^ unveiled 25.03%, 63.86%, 98.07% and − 18.44% of percentages of growth inhibition. These results are summarized in Fig. 3c and admit that **2** and **3** present the highest inhibition percentage (> 60%) on 7G8 *P. falciparum* strains.

From the antimycobacterial tests results (Table 2), it should be noticed that the mixture of FA (A_1_) exhibited a good antitubercular activity with a Minimal Inhibitory Concentration (MIC) value at 9.52 μg mL^−1^. According to Cantrell et al. [47], isolated compounds that exhibit a MIC ≤ 64 μg mL^−1^ are considered promising. For crude extracts, the MIC should be ≤ 125 μg mL^−1^ [48]. The extract made known poor inhibitory activity against *M*ycobacterium *tuberculosis*, exhibiting a MIC and a Minimal Bactericidal Concentration (MBC) of 1250 and 2500 μg mL^−1^ respectively.

**Table 2:** MIC and MBC values of the methanol extract and the mixture of fatty acids (A_1_) against *Mycobacterium tuberculosis* (AC 45)

Plant species	MIC^a^ (μg mL^−1^)	MBC^b^ (μg mL^−1^)	MBC/MIC
*C. batesii*	1250	2500	2
A_1_	9.52	38.25	4
RMP	0.97	7.81	8

*RMP* Rifampicin

^a^Minimum inhibitory concentration

^b^Minimum bactericidal concentration

### Computational results of compound 2

**Fig. 4:**
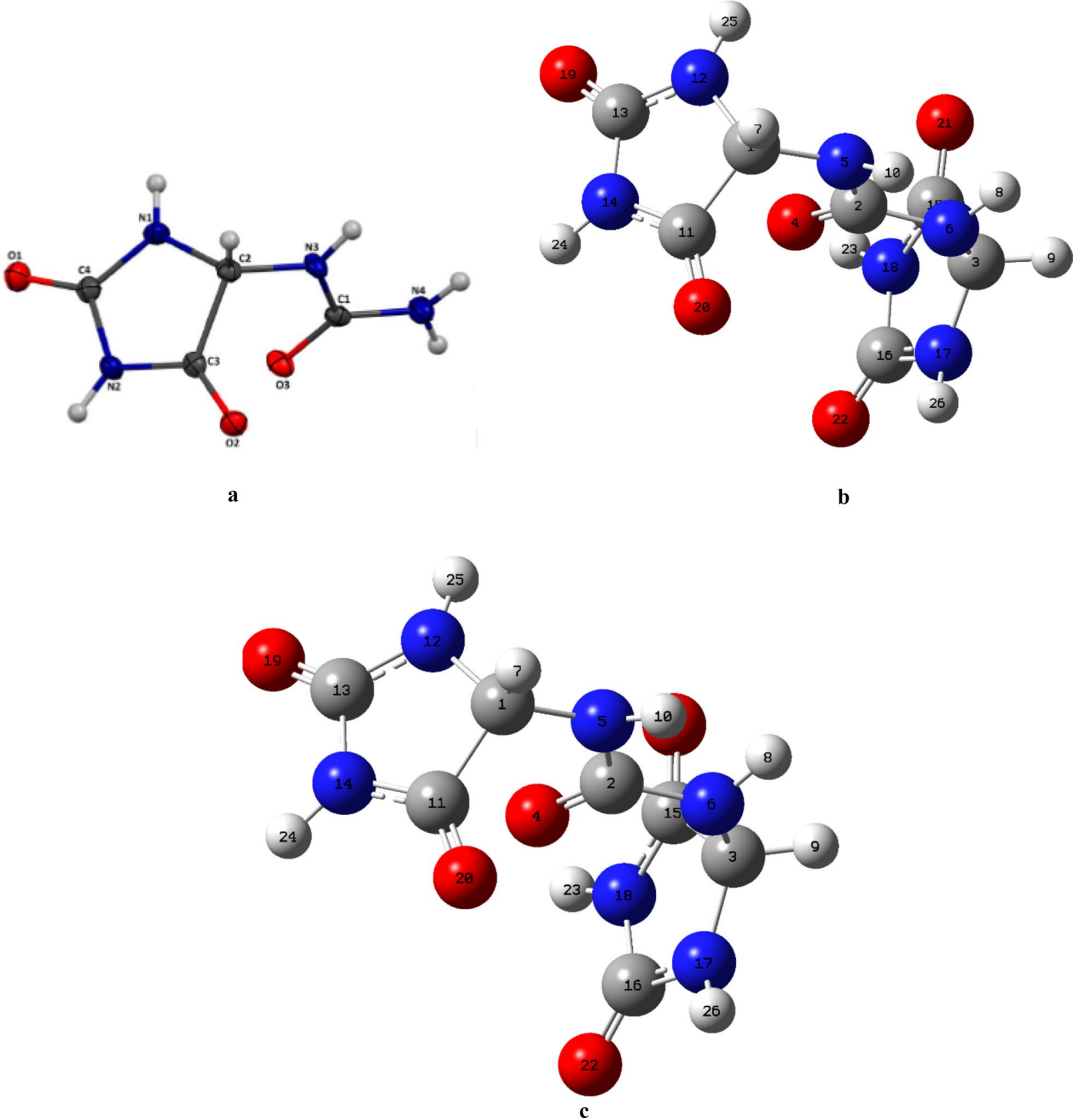
**a** ORTEP diagram of allantoin (**1**) [44]; optimized geometry of batesiin (**2**) at **b** B3LYP/6-31G(d) and **c** B3LYP/6-31++G(d,p)

The structure of compound **2** was assigned based on spectroscopic analyses including, UV, IR, ^1^H- and ^13^C-NMR, 1D and 2D techniques. To get supplementary detailed awareness into the structure, DFT calculations were completed. The structure of the compound with the right stereochemistry was firstly optimized at B3LYP method using 6-31G(d) basis set and the optimized structure was submitted to a relaxed scan around one H–C(5)–N(6)–H dihedral angle. Secondly, an additional relaxed scan around the other H–C(5)–N(6)–H dihedral angle (with no change in basis set) was applied on a conformer with low energy got from the preceding step. The desired conformer still with low energy was thereafter subjected to two relaxed scans around the C(5)–N(6)–C(7)–N(6) dihedral angles. All these scans led to an optimized geometry for **2**, based on the *cis*-relationship between H–C(5)–N(6)–H (^3^* J*(H,H) = 2.0 Hz). It is shown in Fig. 4 and takes in account previous reports on **1** [44, 49]. The five membered ring is almost planar as observed in the case of allantoin (**1**) [19].

**Fig. 5:**
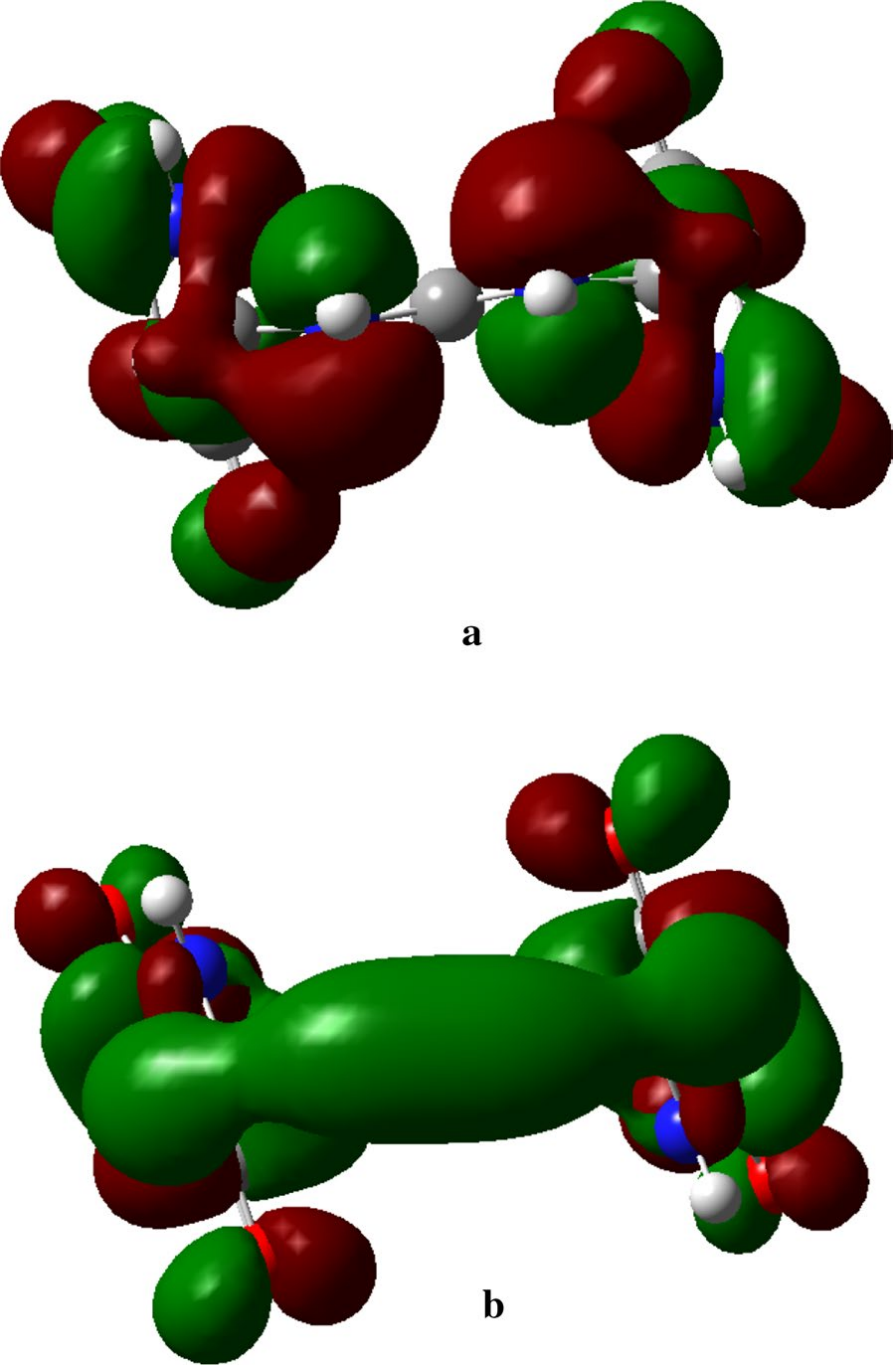
HOMO (**a**) and LUMO (**b**) of **2**, calculated at B3LYP/6-311G++(d,p). The orbitals are plotted at isodensity of 0.02. The green color indicates the positive values of the phase while the negative values are characterized by the red color

The HOMO and LUMO of compound **2** (Fig. 5) as other descriptors were analyzed at B3LYP/6-31G(d) and 6-311G++(d,p) and compared with those of allantoin (**1**). The calculated HOMO–LUMO gap was 6.209 eV; results are summarized in Table 3.

**Table 3:** Some electronic and thermodynamic results of batesiin (**2**) compared to data of allantoin (**1**) at B3LYP

Parameters	Allantoin (**1**) [19]	Batesiin (**2**)	
	B3LYP/6-311G++(d,p)	B3LYP/6-31G(d)	B3LYP/6-311G++(d,p)
SCF energy (a.u.)	− 600.96805371	− 976.275521823	− 976. 58,639,012
Zero point vibrational energy (kcal mol^−1^)	77.961	117.242	116.472
*Rotational constants (GHz)*	1.876	0.713	0.721
	0.876	0.262	0.257
	0.754	0.261	0.254
*Rotational temperatures (Kelvin)*	0.090	0.034	0.035
	0.042	0.013	0.012
	0.036	0.013	0.012
Total energy (thermal) (kcal mol^−1^)	84.462	127.176	126.495
Molar heat capacity at constant volume, c_v_ (cal mol^−1^ K^−1^)	37.756	58.872	59.237
Molar heat capacity at constant pressure, c_p_ (cal mol^−1^ K^−1^)	39.744	60.86	61.225
Total entropy, S (cal mol^−1^ K^−1^)	100.186	129.784	130.265
Total enthalpy, H (kcal mol^−1^)	85.055	127.769	127.088
Frontier MO energies (eV)			
* E*_*LUMO*_	− 1.196	− 0.647	− 1.440
* E*_*HOMO*_	− 7.456	− 7.149	− 7.649
* E*_*LUMO*_–*E*_*HOMO*_	6.260	6.502	6.209
Global reactivity descriptors			
Ionization potential, *IP* (*eV*)	7.456	7.149	7.649
Electron affinity, *EA* (*eV*)	1.196	0.647	1.440
Electronegativity, *χ*	4.326	3.898	4.544
Chemical potential, *μ*	− 4.326	− 3.898	− 4.544
Hardness, *η*	3.130	3.251	3.104
Softness, *S*	0.160	0.154	0.161

The values are calculated in gas phase (at 298.15 K)

The ^1^H- and ^13^C-NMR spectra of compound **2** were experimentally measured in DMSO-*d*_6_ on 500 and 125 MHz spectrometers respectively. The theoretical NMR were calculated at B3LYP/6-31G(d,p) and MPW1PW91/6-31+G(d,p) in DMSO. The chemical shifts were also simulated at B3LYP/6-31G(d), B3LYP/6-31+G(d,p), MPW1PW91/6-31G(d) and MPW1PW91/6-31G(d,p); however, the correlation with the experiment was relatively weak. GIAO (Gauge Invariant Atomic Orbital) formalism was used during these calculations, and the solvent effect was introduced through polarizable continuum model (PCM) by applying integral equation formalism (IEF). A comparison of the theoretical ^13^C-NMR values at B3LYP/6-31G(d,p) and MPW1PW91/6-31+G(d,p) with the experimental ones is given in Table 4. A better correlation with the experiment can be achieved if a scaling factor is applied to the ^13^C-NMR theoretical values.

**Table 4:** Experimental and calculated [at B3LYP/6-31G(d,p) and MPW1PW91/6-31+G(d,p)] ^13^C-NMR data of **1**

Position	^13^C chemical shifts (ppm)		
	Experimental	B3LYP/6-31G(d,p)	MPW1PW91/6-31+G(d,p)
2	156.8	156.1	157.0
4	173.6	174.4	174.6
5	62.4	61.7	62.7
7	157.4	157.0	158.0

**Fig. 6:**
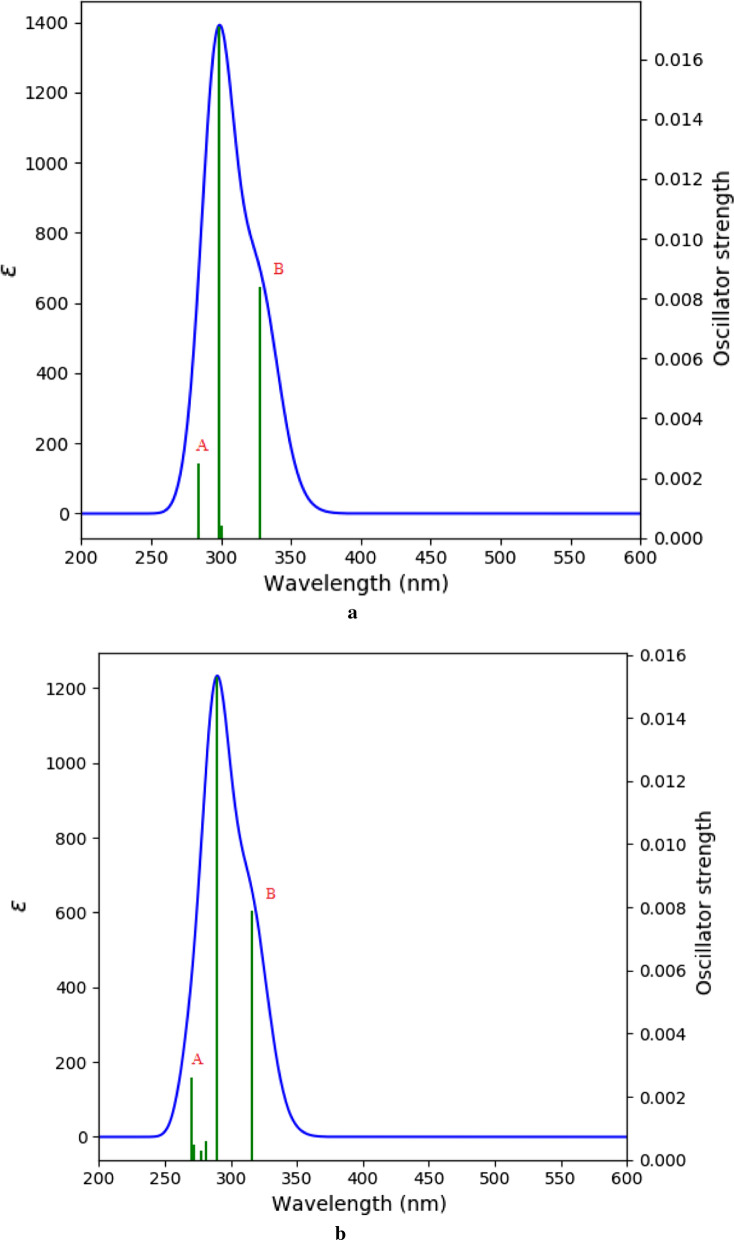
UV–visible spectra of **2** in chloroform (**a**) and in ethanol (**b**), calculated at B3LYP/6-31G+(d,p)

**Fig. 7:**
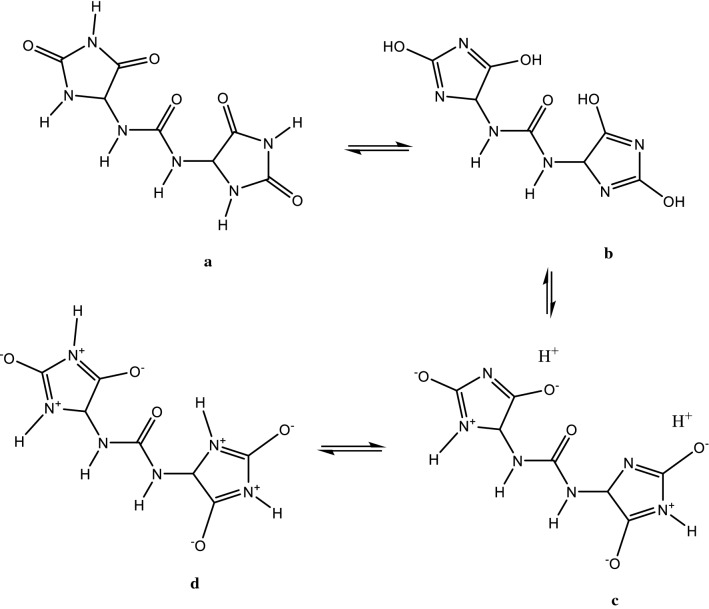
Tautomeric and ionic forms of **2**; based on UV–visible results, it seems most likely that the (**C**) form should be the major one

UV–visible spectrum of **1** displayed three maxima at λ_max_ 183, 195 nm (representing the absorption bands of amide and imide functions) [19] and 265 nm [44]. Despite on the fact that batesiin (**2**) is characterized by the same chromophore groups, its UV–visible spectrum (Fig. 2) exhibits one major absorption band at λ_max_ = 296 nm and minor absorptions as shoulder sections (letters A and B) with λ_max_ around 287 and 304 nm respectively. Simulated UV–visible spectra of **2** were achieved at B3LYP/6-31+G(d,p) with chloroform (non-polar aprotic solvent) and ethanol (polar protic solvent) (Fig. 6), based on its tautomeric and ionic forms (Fig. 7): all λ_max_ results (experimental and theoretical) are summarized in Table 5. Excitation energy (in nm) determined in CHCl_3_ at 283.89 is closed to experimental value at 287 nm while simulation in EtOH exhibits an energy (in nm) at 315.59 which is comparable to the experimental λ_max_ at 304 nm. It must also be precised that results of IR calculations were almost similar to previous ones regarding allantoin (**1**) [19], since **1** and **2** share the same functional groups; hence, there was no need to report them.

**Table 5:** Main experimental and vertical excitation energies of batesiin (**2**) along with oscillator strengths and transitions

Experimental	Excitation energy (nm)		Oscillator strength		Electronic transition	
	CHCl_3_	EtOH	CHCl_3_	EtOH	CHCl_3_	EtOH
287	283.89	270.11	0.0025	0.0026	^a^H–2 → ^b^L + 1	^a^H–2 → ^b^L + 1
296	299.01	289.70	0.0171	0.0153	^a^H–3 → ^b^L	^b^H–3 → ^b^L
304	327.69	315.59	0.0084	0.0079	^a^H–1 → ^b^L	^a^H–1 → ^b^L

^a^H: HOMO

^b^L: LUMO

## Discussion

NMR data summarized in Table 3 strengthen the close relationship between allantoin (**1**) and batesiin (**2**), their ^13^C chemical shifts being almost analogous (Table 1). The main difference relies on ^1^H NMR spectra of both compounds: in **1**, a value at δ_H_ 5.23 (1H, H–C(5)) ppm [50] is equivalent to chemical shift in **2** at δ_H_ 5.30 (2H, d*, *^*3*^* J*(H,H) = 2.0 Hz, H–C(5)) ppm. This 0.7 ppm variation between the aforesaid chemical shifts could be explained by the conformation of **2** (Fig. 4b, c) which seems to induce deshielding of the hydrogen atoms located on carbons C(5).

Results from bioassays against 7G8 *P. falciparum* strain reveal that an increase in concentration (10 to 100 μg mL^−1^) marks an increase in percentages of inhibition for MeOH extract, **2** and **3**, but a decrease (with a negative percentage) for **5**, which should indicate that the latter is totally inactive at high concentrations. The moderate activity of the MeOH extract suggests insufficient synergistic or additive effects of potential antiplasmodial secondary metabolites from *C*. *batesii*. In contrast to the mixture of FA, the weak antimycobacterial properties of the crude extract suggest the occurrence within *C. batesii* of components with very poor antimycobacterial effects (Table 2). Moreover, a report from literature indicates that mycobacteria have a lipid-rich hydrophobic cell wall and are often susceptible to less polar compounds [51]. According to Peterson and Shanholtzer [52], bacteriostatic activity has been defined as a ratio of MBC to MIC of > 4. Hence, essential oil exhibited bacteriostatic activity.

**Fig. 8:**
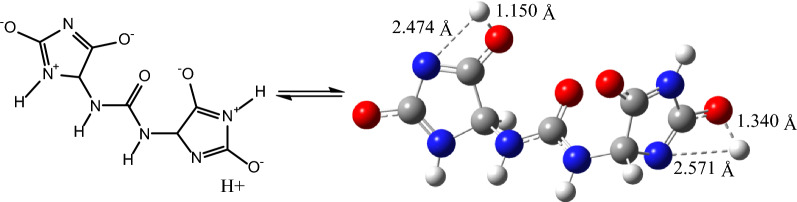
One case of simulated "iminol"/"iminolate" form of **2** with distances between protons and nitrogen or oxygen atoms at B3LYP/6-31G+(d,p); this form seems to induce the electronic transitions (in nm)

**Fig. 9:**
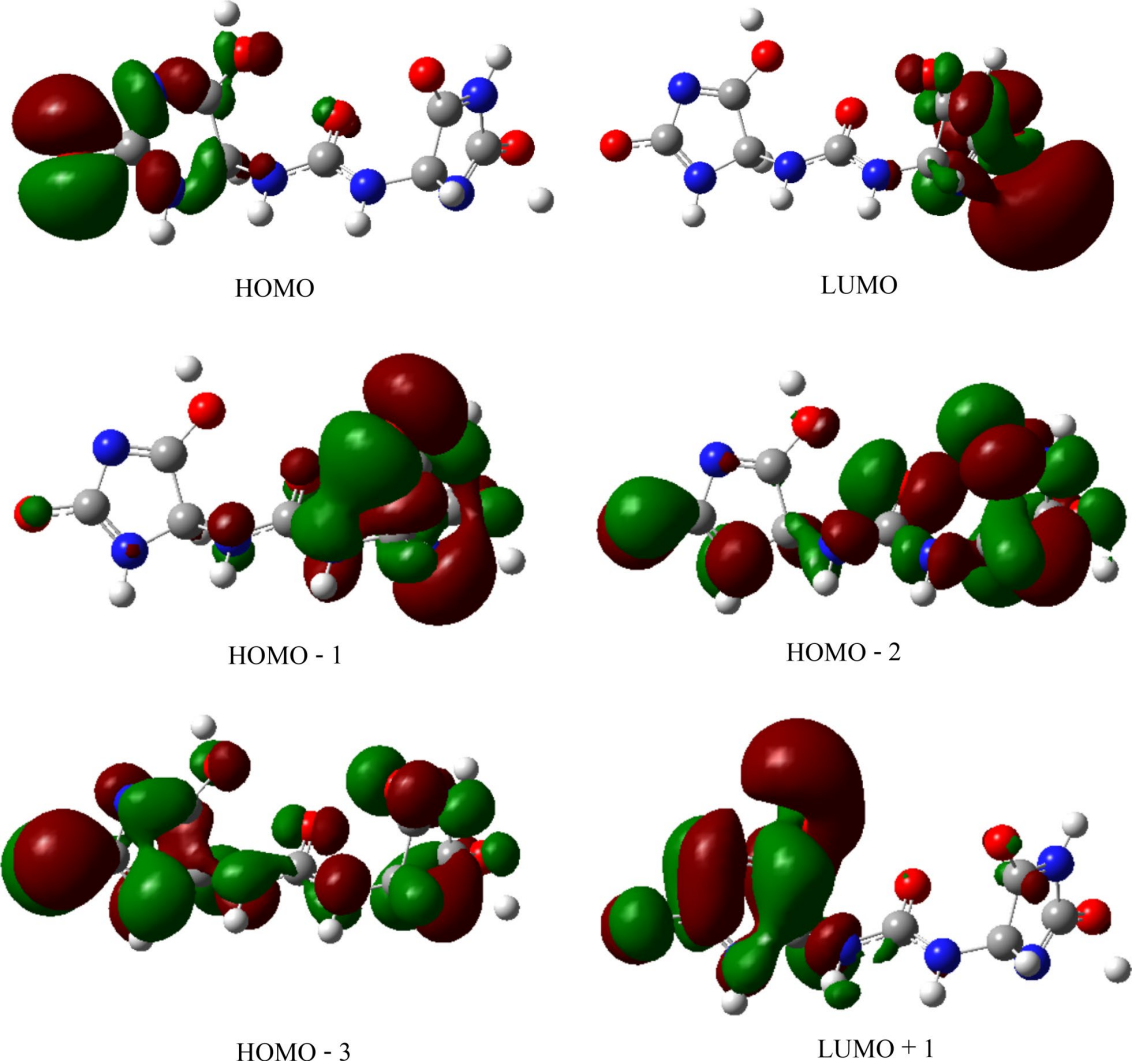
Various MOs of **2** involved in electronic transitions, at B3LYP/6-31G+(d,p). The green color represents the positive values of the phase while the red color is indicative of its negative values

The high value of the HOMO–LUMO band gap is indicative of a relative stability of the molecule towards oxidation–reduction reactions. However, it is less than the HOMO–LUMO band gap of allantoin (**1**) (see Table 3) at the same method, maybe as an expression of additional stability of **2** and difference in biological behavior between both chemical entities. Plots of frontier orbitals show that HOMOs and LUMOs are globally focused over the entire molecule. Meanwhile, in the case of HOMOs, the ureidyl moiety is less concerned by the orbital overlap whereas it is recognized as the area covering the positive phase in LUMOs (Fig. 5). The theoretical ^13^C-NMR values are, on the average, higher than the experimental ones when using MPW1PW91/6-31+G(d,p); by contrast, it is not the same observation with results provided by the B3LYP/6-31G(d,p) process. Regardless of the difference in the absolute values, the theoretical values match nicely with the experimental data. Based on the simulated UV–visible spectrum, batesiin (**2**) should most likely appear as an intermediate between various iminols and iminolates groups, precisely due to additional stability as already hypothesized (Fig. 8). Two free protons are supposed to be located somewhere between, in each case, a nitrogen and an anionic oxygen of the same iminolate group, at a site however nearer to oxygen (distance < 1.4 Ǻ) than to nitrogen (distance > 2.3 Ǻ) (Fig. 8). Hence, a virtual loss of symmetry becomes noticeable within **2**, inducing a change in MOs with an impact on electronic transitions (Fig. 9). LUMO and LUMO + 1 are localized on one imidazole fragment (especially in the region covering a free proton and the anionic oxygen located in its neighborhood) when the other one looks totally unoccupied. Moving from HOMO-1 to HOMO-3 causes an increase in orbital overlap within the entire chemical entity. Table 5 expresses the nature of electronic transitions which are in agreement with the corresponding λ_max_, depending on the nature of the solvent.

## Conclusion

Batesiin (**2**) has been characterized for the first time by means of ^1^H-, ^13^C-NMR and UV spectroscopies; its structure was confirmed by DFT and TD-DFT calculations at B3LYP/6-31G(d,p), 6-31G+(d,p), 6-311G++(d,p) and MPW1PW91/6-31G+(d,p) from this study. The MeOH crude extract of the stems showed a moderate activity against Dd2 *P*. *falciparum* strain with IC_50_ = 50 μg mL^−1^. The antiplasmodial properties of **2** and some other compounds are deduced from high percentages of growth inhibition against the 7G8 *P*. *falciparum* strain; in parallel, antimycobacterial activities against *M*. *tuberculosis* arise from the essential oil equivalent to the mixture of FA (**9**–**14**) with a MIC = 9.52 μg mL^−1^. Additional data corresponding to HOMO, LUMO, enthalpy, entropy or some reactivity descriptors like *IP* or *EA* were also simulated, this time at B3LYP/6-31G(d) and 6-311G++(d,p) and compared with data from literature of allantoin (**1**) [19]; moreover, no comparison with the experiment in this case could be made. This work supports a good agreement between experimental data and DFT calculations in case of structure elucidation. These preliminary results also support the hypothesis of further development of new antimalarial and antitubercular drugs from the genus *Cordia*.

## Methods

### General

Melting points were uncorrected and were measured on a Mettler Toledo instrument. IR spectra were recorded on an Alpha II FT-IR spectrometer from Bruker in the region of 4000–600 cm^–1^, using KBr pellet technique with 1.0 cm^–1^ resolution at room temperature. 1D and 2D NMR spectra were obtained on a Bruker DRX 500 (500 MHz for ^1^H and 125 MHz for ^13^C spectra) spectrometer (Bruker, Rheinstetten, Germany) with chemical shifts reported as δ (ppm), using TMS as an internal standard. The ESI-MS were obtained on LTQ-FT instrument (Thermo Scientific). LC–MS were measured with Shimadzu LC–MS system using a L-column 2 ODS (I.D. 2.1 × 100 mm, Chemical Evaluation and Research Institute, Japan), at a flow rate of 0.2 mL min^−1^ with a detection wavelength of 300 nm and FMW (HCOOH/MeCN/H_2_O = 1:12:87) as eluent, ESI+ 4.5 kV, ESI− 3.5 kV, 250 °C. Optical rotations were measured on a Perkin-Elmer 341 polarimeter. Silica gel 60 (230–400 mesh E. Merck, Darmstadt, Germany) and *Sephadex*^®^ LH-20 were employed for CC, the solvent mixing systems for elution were mainly CH_2_Cl_2_/MeOH for the phytochemical study with increasing polarity and pure MeOH, while precoated aluminum sheets silica gel 60 F254 were used for TLC [53].

### Plant material

The plant material was collected on March 2014 at Koumoul in center region of Cameroon. The identity of plant material was confirmed by the taxonomist Victor Nana. A voucher sample (14,106 SRF) is deposited at the National Herbarium of Cameroon, Yaounde.

### Extraction and isolation

The stems were dried in shade and cut into small pieces and then submitted for further studies. 80% of air dried pieces of stems of *C*. *batesii* (500 g) were extracted with MeOH (5 × 500 mL, 30 min each) using an Elma^®^ sonic S 100 at r. t. The extract was filtered; the filtrate was evaporated to dryness in a Rotavapor. The residue obtained from the MeOH extract (about 53 g) was dissolved into hexane–water 80:20 (100 mL) during 1 day; the resulting hexane–water gum (42 g) was dissolved in a mixture of CHCl_3_–H_2_O 80:20 (100 mL) once again in a period of 24 h; the resulting CHCl_3_–H_2_O extract (36 g) was dissolved in CH_3_COOH–H_2_O 70:30 (100 mL) during 24 h. The final extract (26 g) was submitted to further CC analyses. The crude MeOH extract (26 g) was subjected to CC of LH-20 (2.5 cm, 50 cm, eluent MeOH). Four main fractions were obtained: A (10.36 g), B (4.6 g), C (3.0 g) and D (7.0 g). Fraction A (10.36 g) was subjected to a silica gel CC (1.8 cm, 3 × 50 cm, eluent CH_2_Cl_2_–MeOH 100–1:1) and provided three sub-fraction (A_1_, A_2_, and A_3_). Sub-fraction A_2_ (5.20 g) was fractionated by silica gel CC (1.8 cm, 3 × 50 cm, eluent CH_2_Cl_2_–MeOH 50:1–30:1) to produce two compounds, **4** (4.6 mg) and **5** (11 mg). Fraction B (4.6 g) was chromatographed as described above to give five sub-fractions (B_1_, B_2_, B_3_, B_4_ and B_5_). Sub-fraction B_1_ (0.20 g) was purified by silica gel CC (1.8 cm, 3 × 50 cm, eluent CH_2_Cl_2_–MeOH 30:1–20:1) to yield compound **1** (9 mg). Compound **2** (7.5 mg) was isolated from sub-fraction B_3_ (0.31 g) using a silica gel CC (1.8 cm, 3 × 50 cm, eluent CH_2_Cl_2_–MeOH 40:1–20:1). Sub-fraction B_4_ (0.60 g) was fractionated and purified using a silica gel CC (1.8 cm, 3 × 50 cm, eluent CH_2_Cl_2_–MeOH 40:1–15:1) to provide **3** (4 mg). The essential oil (sub-fraction A_1_, 0.60 g) resulting from the crude extract was analyzed by LC–MS, exhibiting compounds **6**, **7** and **8**. A_1_ was soluble in pure hexane and crystallized from pure CH_2_Cl_2_. 487 mg were analyzed by GC–MS which revealed the compounds **9**, **10**, **11**, **12**, **13** and **14**.

### Characteristics of compound 2

Batesiin (**2**), white solid: mp 231–233 °C. TLC (CH_2_Cl_2_:MeOH, 20:1 v/v) R_f_ = 0.6; ${[\mathrm{\alpha }]}_{\mathrm{D}}^{20}$ = − 98.3° (0.04 M in acetone). ^1^H NMR (500 MHz, DMSO-*d*_6_) δ 10.51 (2H, s, NH), 8.04 (2H, s, NH), 6.88 (2H, d, J = 2 Hz, NH), 5.23 (2H, d, J = 2 Hz); ^13^C NMR (125 MHz, DMSO-*d*_6_) δ 62.4, 156.8, 157.4, 173.6; IR (KBr) cm^–1^ 3425, 3340 (O–H), 3125, 3060 (N–H), 1810, 1740, 1680, 1560 (C=O); UV/Vis: λ_max_ (MeOH) nm: 296. HR-LC/MS (m/z): [M+Na]^+^ calcd for C_7_H_8_N_6_O_5_Na^+^; 279.1608; found 279.1603; analysis (calcd., found for C_7_H_8_N_6_O_5_Na): C (30.12, 30.09); H (2.89, 2.85); N (30.10, 30.10); O (28.66, 28.65).

### Biological assays

#### In vitro cultivation of *P*. *falciparum* strains

*Pf*Dd2 and *Pf*7G8 strains of *P. falciparum* were used in vitro in blood stage culture to test the antimalarial efficacy of MeOH extract of stems of *C*. *batesii* and isolated compounds **2**, **3** and **5**. The culture was maintained at the Laboratory of parasitology, Centre Pasteur du Cameroon. *P. falciparum* culture was maintained according to the method described by Trager and Jensen [54] with slight modifications. *P. falciparum* Dd2 and 7G8 cultures were maintained in fresh O^+ve^ human erythrocytes suspended at 4% haematocrit in RPMI 1640 (Sigma Aldrich—France) containing 0.2% sodium bicarbonate, 0.5% Albumax, 45 μg L^−1^ hypoxanthine and 50 μg L^−1^ gentamicin, and incubated at 37 °C under a gas mixture 5% O_2_, 5% CO_2_, and 90% N_2_. Every day, infected erythrocytes were transferred into fresh complete medium to propagate the culture.

### Drug dilutions

Artemisinin (Sigma Aldrich—France) and isolated compounds were prepared in DMSO. All stocks were then diluted with culture medium to achieve the required concentrations. The final solution of all plant extracts, isolated compounds and artemisinin contained 0.4% DMSO, which was found to be non-toxic to the parasites. Drugs and test compounds were then placed in 96-well flat bottom tissue culture grade plates.

### Assay for antiplasmodial activity

The stems of *C*. *batesii* were evaluated for their antimalarial activity against *P. falciparum* strains Dd2 and 7G8. For drug screening, SYBR green I-based fluorescence assay was setup as described by Smilkstein et al. [46]. Sorbitol synchronized parasites were incubated under normal culture conditions at 2% haematocrit and 1% parasitemia in the absence or presence of increasing concentrations of MeOH extracts of *C*. *batesii*. Artemisinin was used as positive control, while 0.4% DMSO was used as the negative control. After 48 h of incubation, 100 μL of SYBR Green I solution [0.2 μL mL^−1^ of 10,000× SYBR Green I (Sigma Aldrich—France)] in lysis buffer [Tris (20 mM; pH 7.5), EDTA (5 mM), saponin (0.008%; w/v) and Triton X-100 (0.08%; v/v)] was added to each well. The microtiter plate was mixed twice gently with multi-channel pipette and incubated in dark at 37 °C for 1 h. Fluorescence was measured with a fluorescence multi-well plate reader (Perkin Elmer) with excitation and emission wavelength bands centred at 485 and 530 nm, respectively. The fluorescence counts were plotted against the drug concentration and the inhibitory percentage of each plant extract and compound was calculated using the following equation:

$${\text{I}}\left( \% \right) = \frac{{{\text{Abs}}_{{{\text{control}}}} - {\text{Abs}}_{{{\text{extracts}}/{\text{isolated compounds}}}} { }}}{{{\text{Abs}}_{{{\text{control}}}} }} \times 100$$

where Abs_control_ is the absorbance of untreated well and Abs_extracts/isolated compounds_ is the absorbance of extracts or compounds well.

### Antimycobacterial tests

For the present study, the mycobacterium (*M. tuberculosis*) used was a clinical isolated strain resistant to isoniazid codified as AC 45 (this strain was obtained from Sangmelima district’s Hospital in south region of Cameroon). The genetical profile of the resistance has been carried out at Laboratory for Tuberculosis Research (Biotechnology Centre, University of Yaoundé I) through Line probe Assay method. The mycobacteria strains have been cultured at 37 °C for 2 weeks in Middlebrook 7H9 (Himedia, India) supplemented with 0.05% (v/v), 2% glycerol and 10% OADC (oleic acid-albumin-dextrose-catalase of Liofilchem s.r.l, Italia). The optical density of 0.45 to 0.55 was measured using spectrophotometer at 550 nm to obtain a suspension of 1.5 × 108 UFC mL^−1^. The activity of all phytochemicals (extract and pure compounds) against the aforementioned *M. tuberculosis* strains was tested using the microplate Alamar Blue assay as described previously by Collins and Franzblau [55]. In a 96 well microplates, all wells received 100 μL of supplemented Middlebrook 7H9 broth, then working metabolites solutions (100 μL) were poured into the first well of each row, from which twofold dilution series were made through the microplate column. The test inoculum (100 μL) was added to all testing wells, as well as to the drug-free control wells. The final concentration of DMSO in wells was 7% v/v. The final concentrations tested ranged from 250 to 0.244 μg mL^−1^ for pure compounds and 5000 to 4.882 μg mL^−1^ for extracts. Rifampicin was used as standard drug. Each concentration was assayed in triplicate. Each microplate was sealed with parafilm paper and incubated for 14 days at 37 °C. After that, 40 μL of Alamar blue solution was added to two columns of each triplicate in order to show mycobacterial growth and the plates were re-incubated at 37 °C for 24 h. After 1 day of incubation, the MIC was defined as the lowest concentration of phytochemicals that inhibited the bacterial growth (prevents a color change from blue to pink) after incubation time [56]. For the MBC determination, 50 μL of each well which concentration was ≥ MIC was sub-cultured in 150 μL of Mbk 7H9 medium and incubated at 37 °C for 10 days, then mycobacterial growth was carried out by addition of 40 μL of alamar blue. MBC was defined as the lowest concentration of extract at which no visible growth of the germ was observed.

### Computational details

All calculations were performed with Gaussian 09 suite of programs [57] and UV–visible curves were generated by GaussSum [58]. Geometries were optimized at hybrid B3LYP method using 6-31G(d) basis set. The B3LYP mode provides a good balance between cost and precision [59, 60]. A preliminary predict of the geometry of compound **2** is based on the stereochemistry ascribed through 1D and 2D NMR characterizations. Compound **2** has conformational flexibility around the symmetric dihedral angles H–C(5)–N(6)–H and C(5)–N(6)–C(7)–N(6). The dihedral angles around the C(5)–N(6) axes were scanned at 15 degrees step and a minimum at 22.9° around the C(5)–N(6) axes was retained because of its conformation closed to the structure of allantoin (**1**). Afterwards, dihedral angles around the N(6)–C(7) axes were scanned at 10 degrees step, to find the lowest energy conformer. It was then submitted to geometry optimization at B3LYP/6-31G(d) and B3LYP/6-311++G(d,p) levels of theory to provide the optimized geometries of **2** (Fig. 4). The optimized structure was in each case confirmed by frequency analysis at the same levels as a true minimum (no imaginary frequency). Six methods were evaluated for the simulation of ^1^H- and ^13^C-NMR spectra; B3LYP/6-31G(d), B3LYP/6-31G(d,p), B3LYP/6-31G+(d,p), MPW1PW91/6-31G(d), MPW1PW91/6-31G(d,p) and MPW1PW91/6-31G+(d,p). The electronic properties at isodensity 0.02 such as Ionization Potential (*IP*), Electron Affinity (*EA*), HOMO, LUMO and band gaps were calculated at B3LYP/6-31G(d) and B3LYP/6-311G++(d,p). The band gap was taken as the difference in energies of HOMO and LUMO. Meantime, thermodynamic properties like enthalpy, entropy or molar capacity at constant volume along with reactivity descriptors like chemical potential, electronegativity, hardness, softness were also calculated by means of the same methods. TD-DFT studies were evaluated at B3LYP/6-31G+(d,p) in CHCl_3_ and in EtOH after a geometry optimization at the same level of theory of the iminolate in Fig. 8. Six steps were applied to get excitation energies.

## Supplementary Information

**Additional file 1.** NMR spectra of compound **2**, some HMBC correlations, GC–MS analyses of compounds **9**, **10**, **11**, **12**, **13** and **14**, LC–MS and GC–MS chromatograms of the extract of *C*. *batesii*, the ESI-MS spectrum of compound **2**, the genotype profile of *M. tuberculosis* codified AC 45, the shielding tensors of the nuclei of **2**, the electronic (HOMO, LUMO) properties, the cartesian coordinates of the optimized geometry of compound **2** and TD-DFT are available as additional file.

## Data Availability

All data generated or analyzed during this study are included in this published article (and its Additional file 1).

## References

[1] World Health Organization. Accelerating progress on HIV, tuberculosis, malaria, hepatitis and neglected tropical diseases: a new agenda for 2016–2030. 2015. World Health Organization. https://apps.who.int/iris/handle/10665/204419. Accessed 15 Sept 2020.

[2] Rasmussen C, Nyunt MM, Ringwald P (2017). Artemisinin-resistant *Plasmodium falciparum* in Africa. N Engl J Med.

[3] Houben RM, Dodd PJ (2016). The global burden of latent tuberculosis infection: a re-estimation using mathematical modelling. PLoS Med.

[4] Der Meeren OV, Hatherill M, Nduba V, Wilkinson RJ, Muyoyeta M, Van Brakel E (2018). Phase 2b controlled trial of M72/AS01E vaccine to prevent tuberculosis. N Engl J Med.

[5] World malaria report. Geneva: World Health Organization; 2019. Licence: CC BY-NC-SA 3.0 IGO.

[6] Romero JA, Acosta ME, Gamboa ND, Mijares MR, De Sanctis JB, Llovera LJ, Charris JE (2019). Synthesis, antimalarial, antiproliferative, and apoptotic activities of benzimidazole-5-carboxamide derivatives. Med Chem Res.

[7] Al-Otaibi JS, Almuqrin AH, Mary YS, Thomas R (2020). Modeling the conformational preference, spectroscopic properties, UV light harvesting efficiency, biological receptor inhibitory ability and other physico-chemical properties of five imidazole derivatives using quantum mechanical and molecular mechanics tools. J Mol Liq.

[8] Lodewyk MW, Siebert MR, Tantillo DJ (2012). Computational Prediction of ^1^H and ^13^C chemical shifts: a useful tool for natural product, mechanistic, and synthetic organic chemistry. Chem Rev.

[9] Oza MJ, Kulkarni YA (2017). Traditional uses, phytochemistry and pharmacology of the medicinal species of the genus *Cordia* (Boraginaceae). J Pharm Pharmacol.

[10] Barroso ICE, de Oliveira F, Ciarelli DM (2009). Morphology of the dispersion unit and germination of *Cordia sellowiana* and *Cordia myxa*. Bragantia.

[11] Ioset JR, Marston A, Gupta MP, Hostettmann K (2000). Antifungal and larvicidal compounds from the root bark of *Cordia alliodora*. J Nat Prod.

[12] Marston A, Zagorski MG, Hostettmann K (1988). Antifungal polyphenols from *Cordia goetzei*. Helv Chim Acta.

[13] Hernandez T, Canales M, Teran B, Avila O, Duran A, Garcia AM (2007). Antimicrobial activity of the essential oil and extracts of *Cordia curassavica* (Boraginaceae). J Ethnopharmacol.

[14] Okusa PN, Penge O, Devleeschouwer M, Duez P (2007). Direct and indirect antimicrobial effects and antioxidant activity of *Cordia gilletii* De Wild (Boraginaceae). J Ethnopharmacol.

[15] Jamkhande PG, Ghante MH, Barde SR, Ajgunde BR (2019). Antimycobacterial, antimicrobial, antioxidant activities and *in silico* PASS investigations of root fractions and extract of *Cordia dichotoma*. Orient Pharm Exp Med.

[16] Tracey MV, Paech K, Tracey MV (1955). Urea and ureides. Modern methods of plant analysis.

[17] da Silva VC, de Carvalho MG, Alves AN (2008). Chemical constituents from leaves of *Palicourea coriacea* (Rubiaceae). J Nat Med.

[18] Kus N, Bayar SH, Fausto R (2009). Thermal decomposition of allantoin as probed by matrix isolation FTIR spectroscopy. Tetrahedron.

[19] Alam MJ, Ahmad S (2015). FTIR, FT-Raman, UV–visible spectra and quantum chemical calculations of allantoin molecule and its hydrogen bonded dimers. Spectrochim Acta A.

[20] Nariya PB, Shukla VJ, Acharya RN, Nariya MB, Dhalani JM, Patel AS, Ambasana PA (2018). Triterpenoid and fatty acid contents from the stem bark of *Cordia dichotoma*. Folia Med (Plovdiv).

[21] McKinney JD, Höner zu Bentrup K, Muñoz-Elías EJ, Miczak A, Chen B, Chan WT, Swenson D, Sacchettini JC (2000). Persistence of *Mycobacterium tuberculosis* in macrophages and mice requires the glyoxylate shunt enzyme isocitrate lyase. Nature.

[22] Arai M, Yamano Y, Kamiya K, Setiawan A, Kobayashi M (2016). Anti-dormant mycobacterial activity and target molecule of melophlins, tetramic acid derivatives isolated from a marine sponge of *Melophlus* sp. J Nat Med.

[23] Lawal TO, Mbanu AE, Adeniyi BA (2014). Inhibitory activities of *Ceiba**pentandra* (L.) Gaertn and *Cordia**sebestena* Linn. on selected rapidly growing mycobacteria. Afr J Microbiol Res.

[24] Dai J, Sorribas A, Yoshida WY, Williams PG (2010). Sebestenoids A-D, BACE1 inhibitors from *Cordia sebestena*. Phytochemistry.

[25] Rekha M, Kowsalya M (2020). A new organic dye *Cordia sebestena* sensitized solar cell with current-voltage characteristics. Asian J Chem.

[26] Aayisha S, Devi TSR, Janani S, Muthu S, Raja M, Sevvanthi S (2019). DFT, molecular docking and experimental FT-IR, FT-Raman, NMR inquisitions on “4-chloro-*N*-(4,5-dihydro-1*H*-imidazol-2-yl)-6-methoxy-2-methylpyrimidin-5-amine”: alpha-2-imidazoline receptor agonist antihypertensive agent. J Mol Struct.

[27] Zhou CX, Jian-Xia Mo JX, Wang XY, Zhang J, Gan LS (2011). Theoretical study on flueggenines A and B: a comparison of calculated spectroscopic properties with IR, UV and ECD experimental data. J Mol Struct.

[28] Becke DA (1993). Density-functional thermochemistry. III. The role of exact exchange. J Chem Phys.

[29] Lee C, Yang W, Parr RG (1988). Development of the Colle-Salvetti correlation-energy formula into a functional of the electron density. Phys Rev B.

[30] Adamo C, Barone V (1998). Exchange functionals with improved long-range behavior and adiabatic connection methods without adjustable parameters: the *m*PW and *m*PW1PW models. J Chem Phys.

[31] Perdew JP, Chevary JA, Vosko SH, Jackson KA, Pederson MR, Singh DJ, Fiolhais C (1992). Atoms, molecules, solids, and surfaces: applications of the generalized gradient approximation for exchange and correlation. Phys Rev B.

[32] Zeukang RD, Noundou XS, Fotsing MT, Kuiate TT, Mbafor JT, Krause RWM (2019). Cordidepsine is a potential new anti-HIV depsidone from *Cordia millenii*. Molecules.

[33] Li P, Feng ZX, Ye D, Huan W, Gang WD, Dong LX (2003). Chemical constituents from the whole plant of *Euphorbia altotibetic*. Helv Chim Acta.

[34] Velde VV, Lavie D, Zelnik R, Matida AK, Panizza S (1982). Cordialin A and B, two new triterpenes from *Cordia verbenacea*. J Chem Soc Perkin Trans.

[35] Formica JV, Regelson W (1995). Review of the biology of quercetin and related bioflavonoids. Food Chem Toxicol.

[36] Hergert HL (1956). The flavonoids of lodgepole pine bark. J Org Chem.

[37] Coughlin JL, Winnik B, Buckley B (2011). Measurement of bisphenol A, bisphenol A β-D-glucuronide, genistein, and genistein 4′-β-D-glucuronide via SPE and HPLC–MS/MS. Anal Bioanal Chem.

[38] Deliy IV, Vlasova EN, Nuzhdin AL, Gerasimov EY, Bukhtiyarova GA (2014). Hydrodeoxygenation of methyl palmitate over sulfided Mo/Al_2_O_3_, CoMo/Al_2_O_3_ and NiMo/Al_2_O_3_ catalysts. RSC Adv.

[39] Gao H, Hong K, Zhang X, Liu HW, Wang NL, Zhuang L, Yao XS (2007). New steryl esters of fatty acids from the mangrove fungus *Aspergillus awamori*. Helv Chim Acta.

[40] El-Shouny WA, Ali SS, Sun J, Samy SL, Ali A (2018). Drug resistance profile and molecular characterization of extended spectrum beta-lactamase (ESβL)-producing *Pseudomonas aeruginosa* isolated from burn wound infections; essential oils and their potential for utilization. Microb Pathog.

[41] Kazi SA, Clark P, Campi EM, Jackson WR, Hearn MTW (2019). Metathesis reactions with a low-cost spinning disk system. Green Chem Lett Rev.

[42] Azuma CM, dos Santos FCS, Lago JHG (2011). Flavonoids and fatty acids of *Camellia japonica* leaves extract. Braz Pharmacogn.

[43] Nie Y & Stürzenbaum SR. Model nematodes. In: Obesity research in animal models for the study of human disease. 2nd edn. Amsterdam: Elsevier Inc; 2017. p. 267–80.

[44] Lakshmanan G, Sivaraj C, Ammar A, Krishnan DA, Gopinath S, Saravanan K (2019). Isolation and structural elucidation of allantoin, a bioactive compound from *Cleome viscosa*: a combined experimental and computational investigation. Pharmacogn J.

[45] Fulmer GR, Miller AJM, Sherden NH, Gottlieb HE, Nudelman A, Stoltz BM (2010). NMR chemical shifts of trace impurities: common laboratory solvents, organics, and gases in deuterated solvents relevant to the organometallic chemist. Organometallics.

[46] Smilkstein M, Sriwilaijaroen N, Kelly JX, Wilairat D, Riscoe M (2004). Simple and inexpensive fluorescence based technique for high-throughput antimalarial screening. Antimicrob Agents Chemother.

[47] Cantrell CL, Franzblau SG, Fischer NH (2001). Antimycobacterial plant terpenoids. Planta Med.

[48] Gu JQ, Wang Y, Franzblau SG, Montenegro G, Yang D, Timmermann BN (2004). Antitubercular constituents of *Valeriana laxiflora*. Planta Med.

[49] Mootz D (1965). The crystal structure of DL-allantoin. Acta Cryst.

[50] Asia NR, Fatma UA, Mayades S, Mutasem OT (2004). Investigation of the active constituents of *Portulaca oleraceae* (Portulacaceae) growing in Jordan. Pak J Pharm Sci.

[51] Pauli GF, Case RJ, Inui T, Wang Y, Cho S, Fischer NH, Franzblau SG (2005). New perspectives on natural products in TB drug research. Life Sci.

[52] Peterson LR, Shanholtzer CJ (1992). Tests for bactericidal effects of antimicrobial agents: technical performance and clinical relevance. Clin Microbiol Rev.

[53] Tiam ER, Bikobo SDN, Zintchem AAA, Nyemeck NM, Ndedi EDFM, Diboué PHB (2019). Secondary metabolites from *Triclisia gilletii*(Menispermaceae) withantimycobacterial activity against *Mycobacterium tuberculosis*. Nat Prod Res.

[54] Trager W, Jensen JB (1976). Human malaria parasites in continuous culture. Science.

[55] Collins L, Franzblau SG (1997). Microplate alamar blue assay versus BACTEC 460 system for high-throughput screening of compounds against *Mycobacterium tuberculosis* and *Mycobacterium avium*. Antimicrob Agents Chemother.

[56] CLSI (2011). Susceptibility testing of Mycobacteria, Nocardiae, and other aerobic actinomycetes–second edition, Approved Standard M24–A2.

[57] Frisch MJ, Trucks GW, Schlegel HB, Scuseria GE, Robb MA, Cheeseman JR (2010). Gaussian 09, Revision B.01.

[58] O’Boyle NM, Tenderholt AL, Langner KM (2008). J Comp Chem.

[59] Mirzaei M, Elmi F, Hadipour NL (2006). A systematic investigation of hydrogen-bonding effects on the ^17^O, ^14^N, and ^2^H nuclear quadrupole resonance parameters of anhydrous and monohydrated cytosine crystalline structures: a density functional theory study. J Phys Chem B.

[60] Behzadi H, Hadipour NL, Mirzaei M (2007). A density functional study of ^17^O, ^14^N and ^2^H electric field gradient tensors in the real crystalline structure of α-glycine. Biophys Chem.

[61] Coxon B, Fatiadi AJ, Sniegoski LT, Hertz HS, Schaffer R (1977). A novel acylative degradation of uric acid, Carbon-13 nuclear magnetic resonance studies of uric acid and its degradation products. J Org Chem.

