# Supplementary material for: Experimental and computational studies of an antiplasmodial derivative of allantoin; antimycobacterial essential oil from Cordia batesii WERNHAM (Boraginaceae)
Source: BMC Chem. 2021 Mar 5;15(1):15. doi: 10.1186/s13065-021-00742-5 (PMC7934435; doi:10.1186/s13065-021-00742-5)
Supplement: Supplementary file 1 — Additional file 1. NMR spectra of compound 2, some HMBC correlations, GC–MS analyses of compounds 9, 10, 11, 12, 13 and 14, LC–MS and GC–MS chromatograms of the extract of C. batesii, the ESI-MS spectrum of compound 2, the genotype profile of M. tuberculosis codified AC 45, the shielding tensors of the nuclei of 2, the electronic (HOMO, LUMO) properties, the cartesian coordinates of the optimized geometry of compound 2 and TD-DFT are available as additional file. [file 13065_2021_742_MOESM1_ESM.pdf]

Additional file 1

Experimental and computational studies of an antiplasmodial derivative of allantoin; antimycobacterial essential oil from *Cordia batesii* WERNHAM (Boraginaceae)

Eric Robert Tiam[1], Dominique Serge Ngono Bikobo[1], Ibrahim Mbouombouo Ndassa[2,3], Norbert Mbabi Nyemeck II[1], Auguste Abouem A Zintchem[1,3*], Lawrence Ayong[4], Patrick Hervé Betote Diboué[5], Bruno Lenta Ndjakou[1,3], Joséphine Ngo Mbing[1†], Dieudonné Emmanuel Pegnyemb[1†].

Corresponding author:

Dr Auguste Abouem A Zintchem, Higher Teachers Training College, Department of Chemistry, University of Yaoundé I, P.O Box 47, Yaounde, Cameroon. E-mail: augabouem@yahoo.fr
Phone: +237 699 73 22 16.

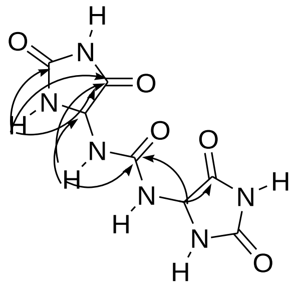

Fig. 1S. Selected HMBC correlations of compound **2**

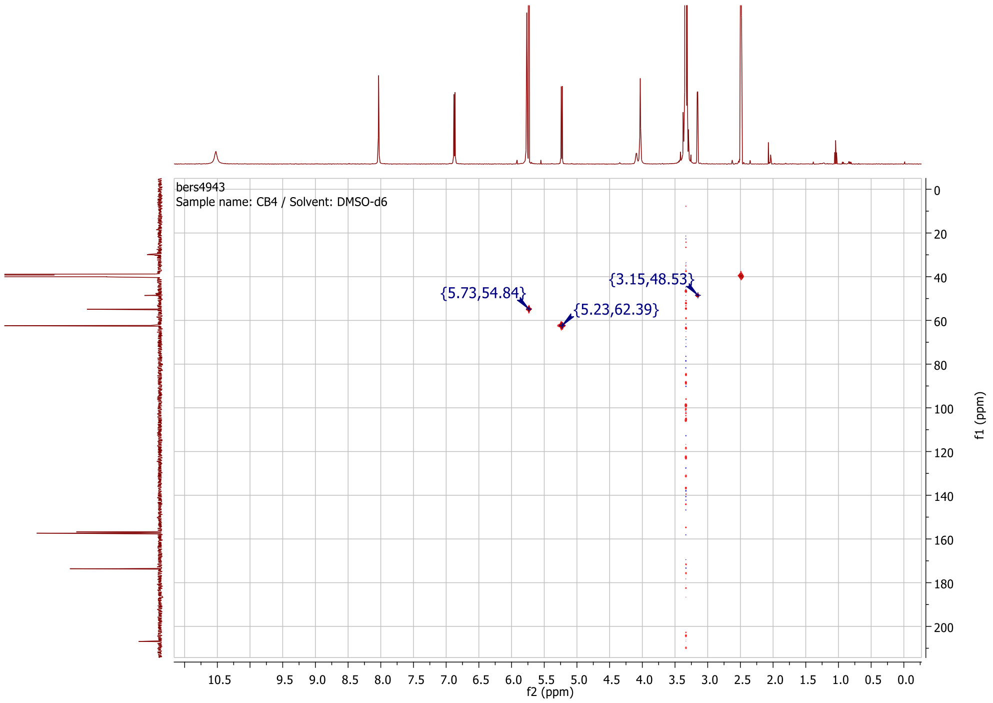

Fig. 2S. HSQC spectrum of compound **2**

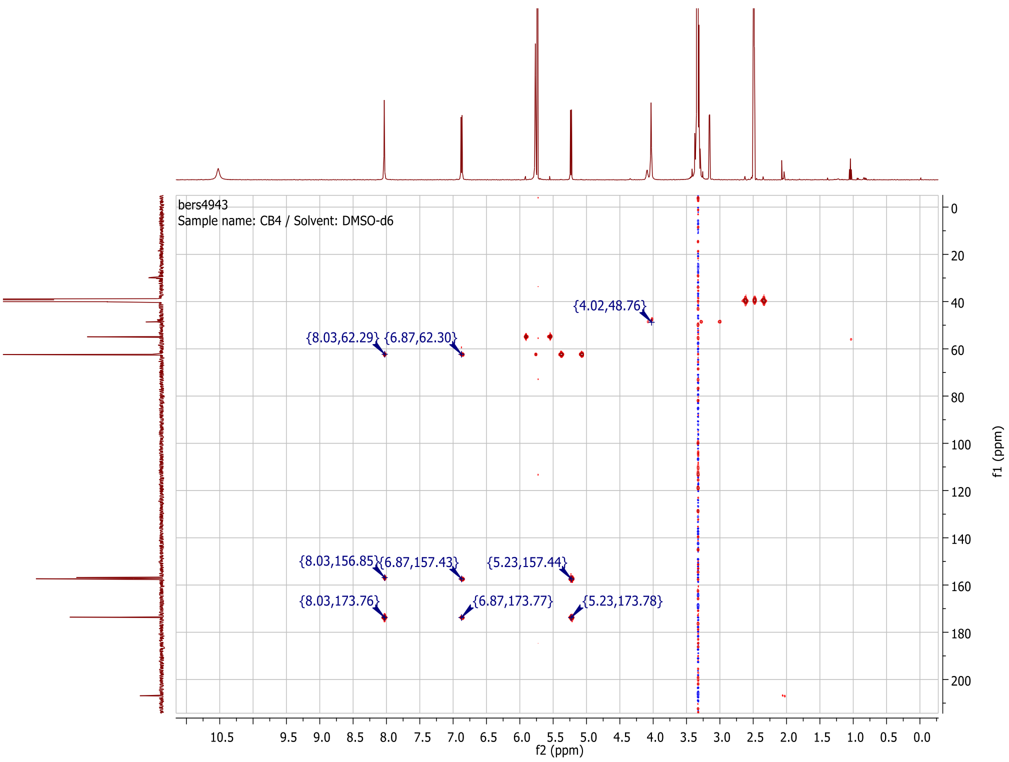

Fig. 3S. HMBC spectrum of compound **2**

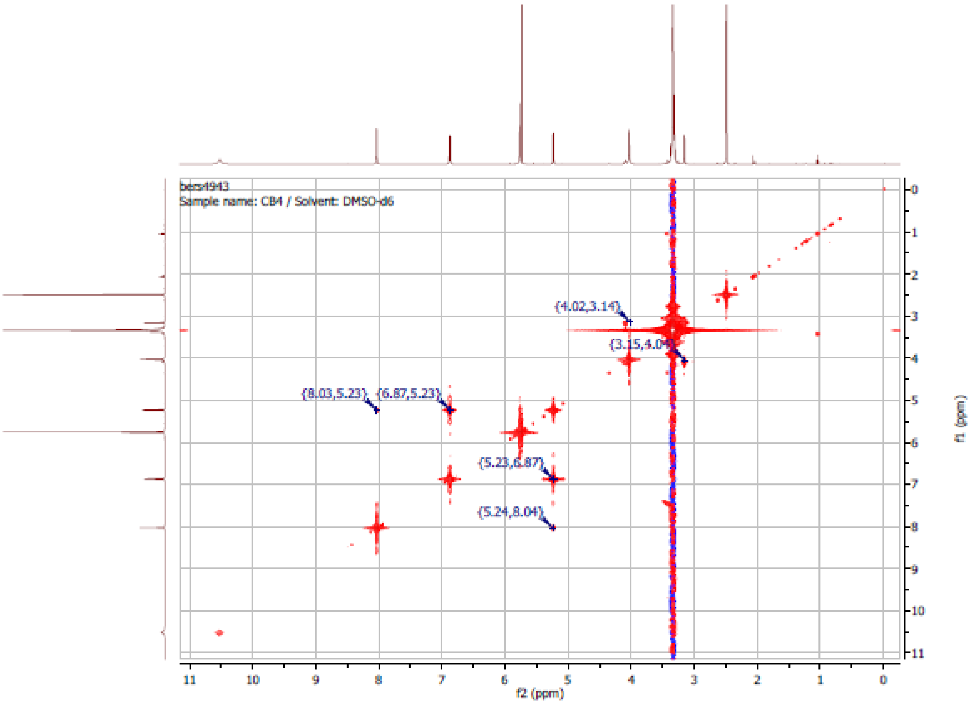

Fig. 4S. ^{1}H - ^{1}H COSY spectrum of compound **2**

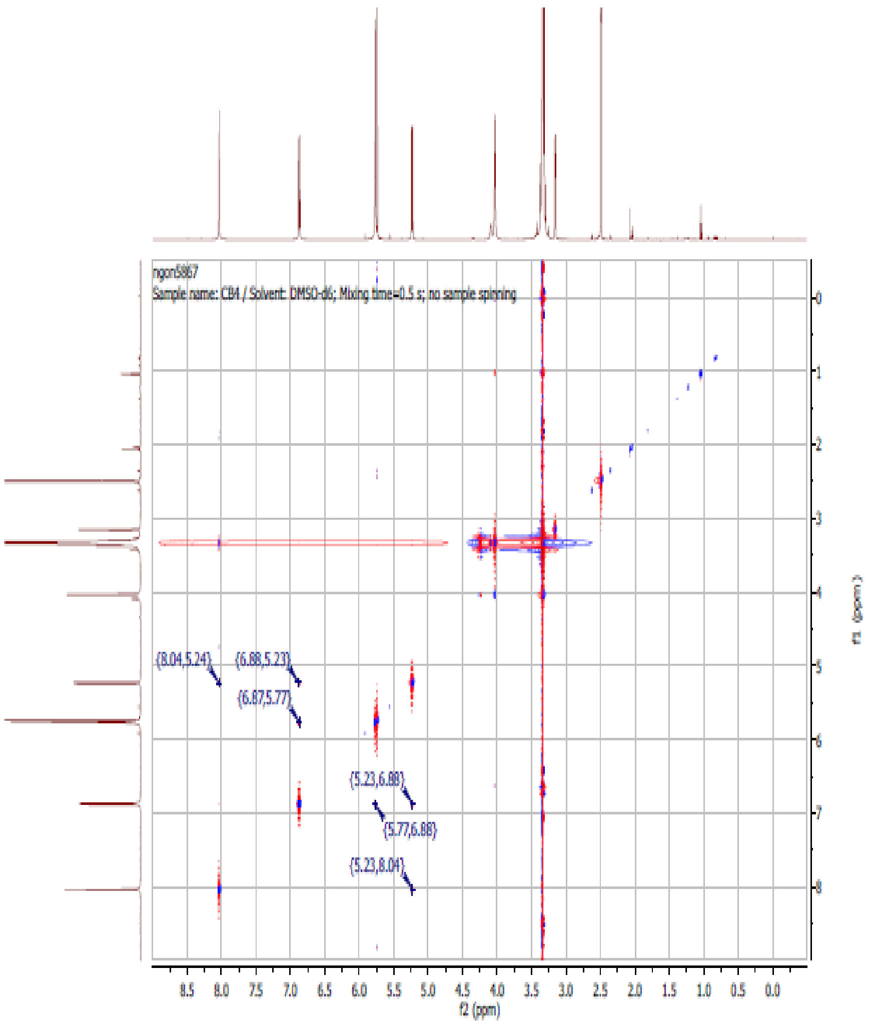

Fig. 5S. NOESY spectrum of compound **2**

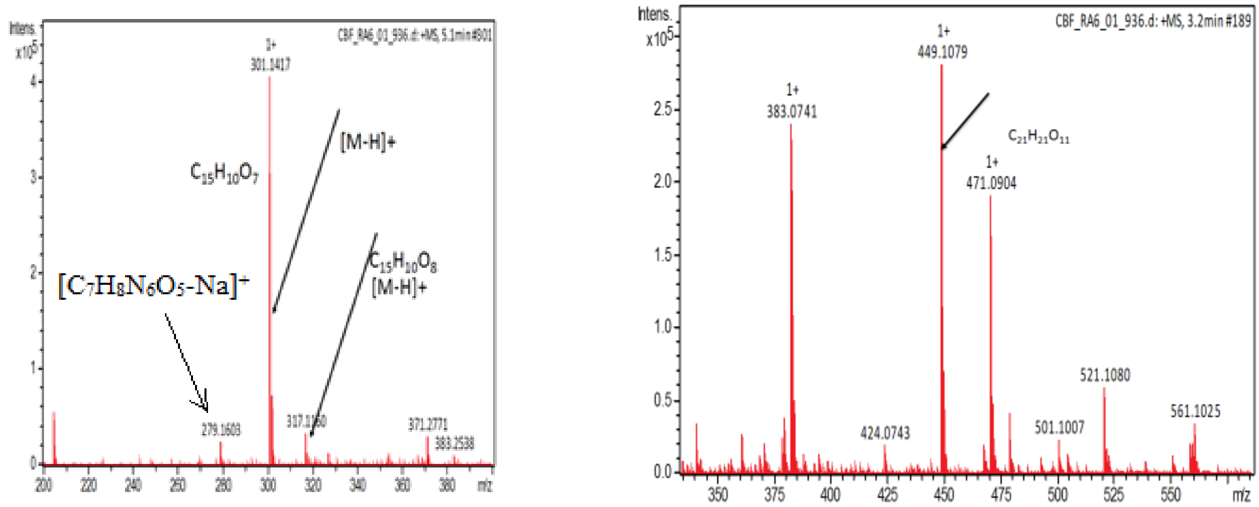

Fig. 6S: LC–MS chromatogram obtained from *C. batesii* (Compounds **6**, **7** and **8**)

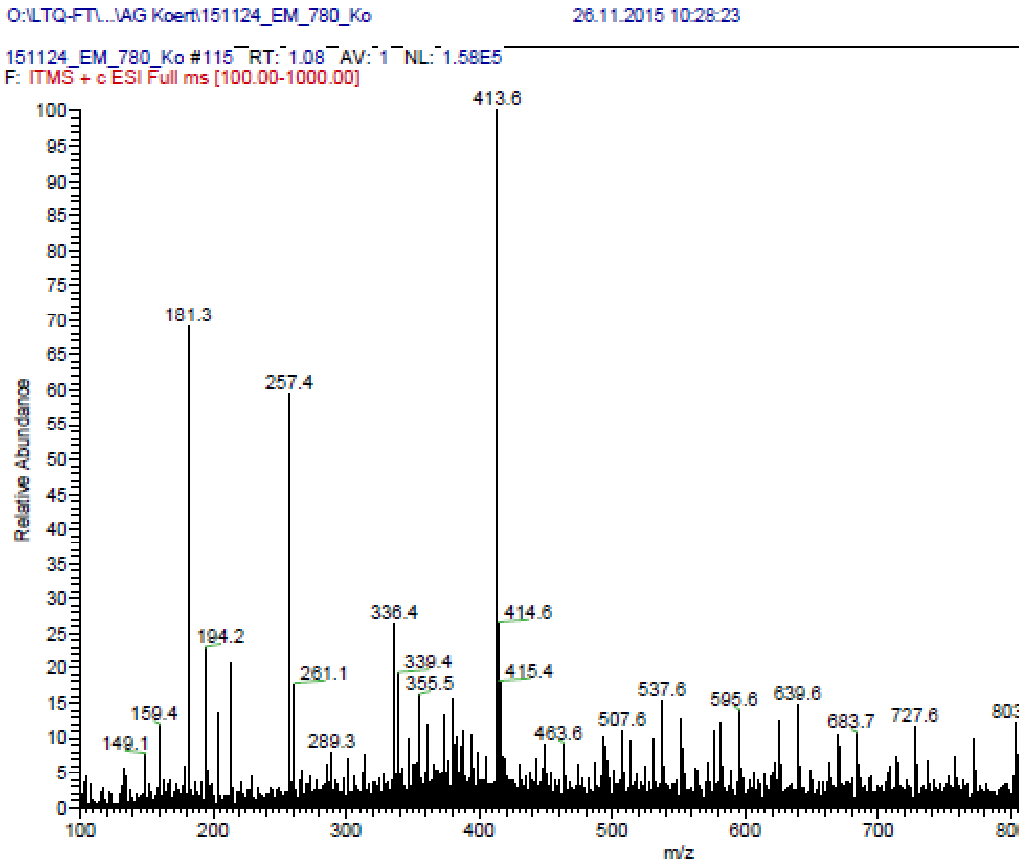

Fig. 7S. ESI-MS spectrum of compound **2**

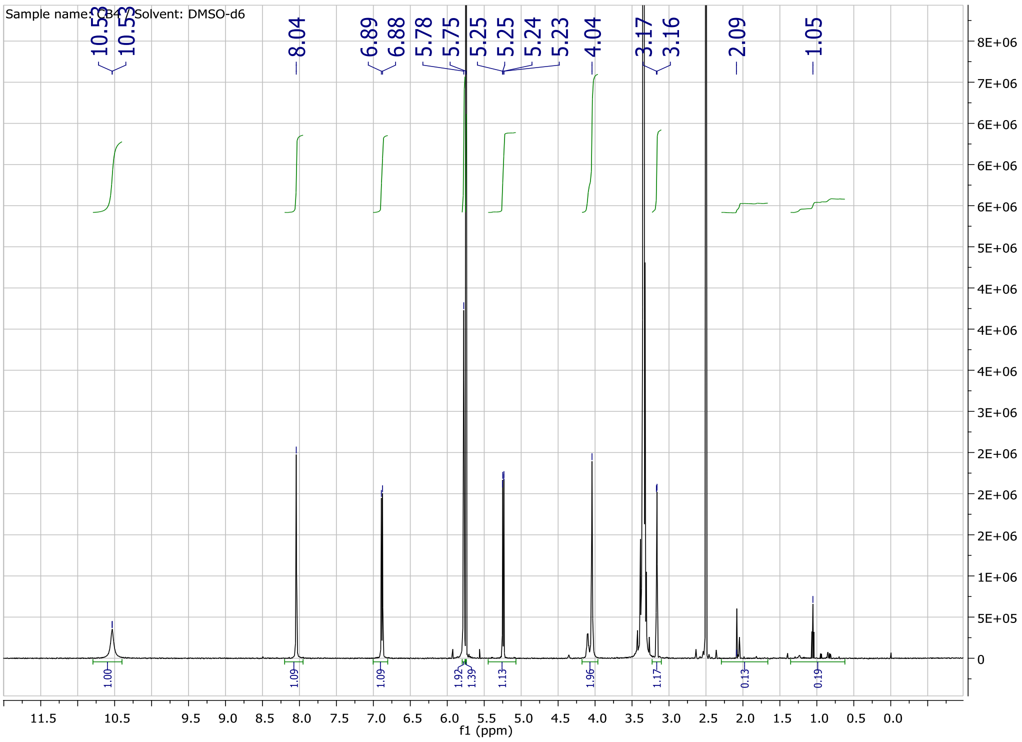

Fig. 8S. ^{1}H–NMR spectrum of compound **2**

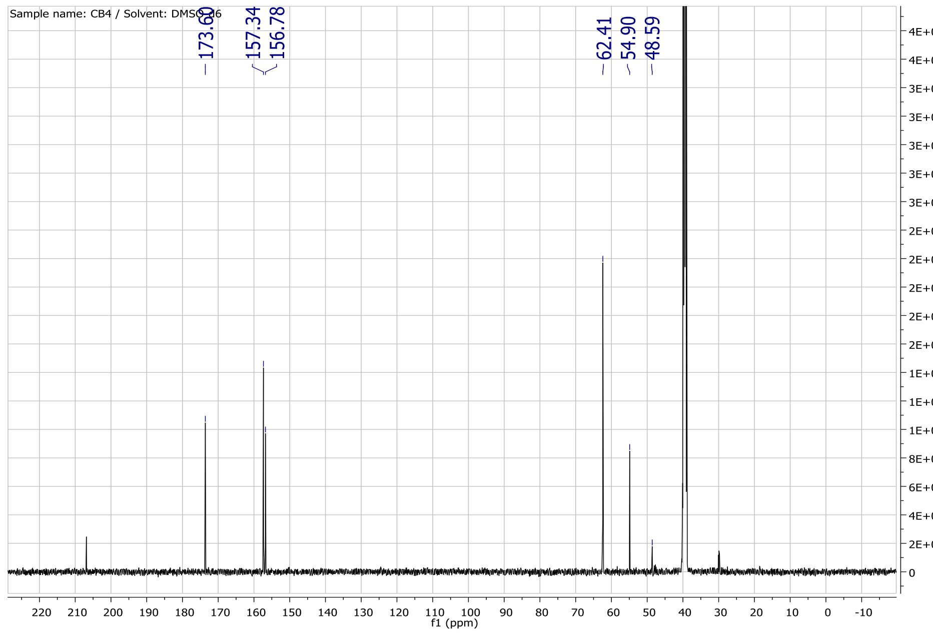

Fig. 9S. ^{13}C–NMR spectrum of compound **2**

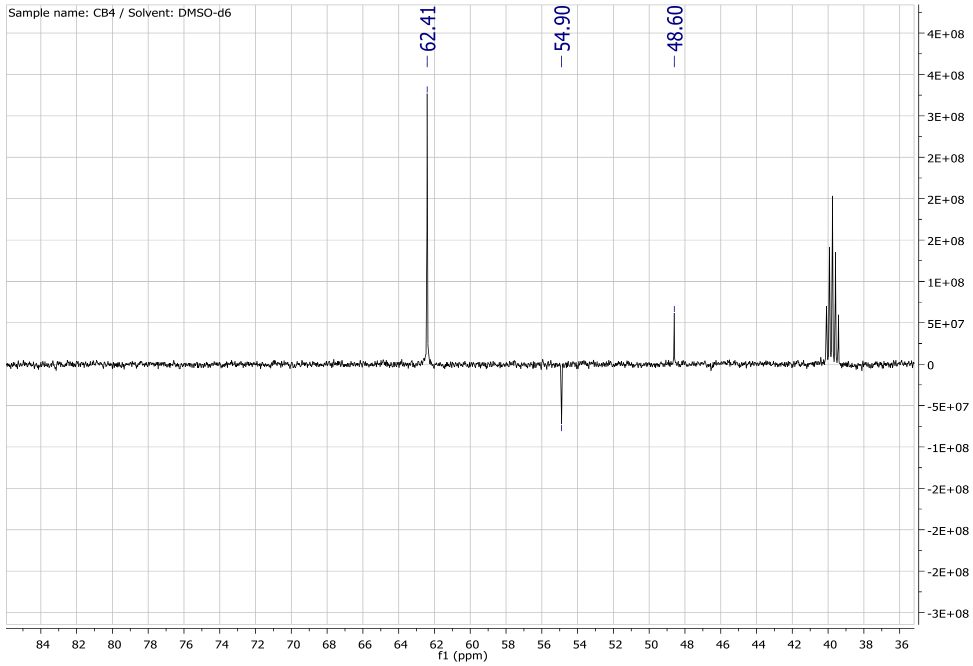

Fig. 10S. DEPT 135 spectrum of compound **2**

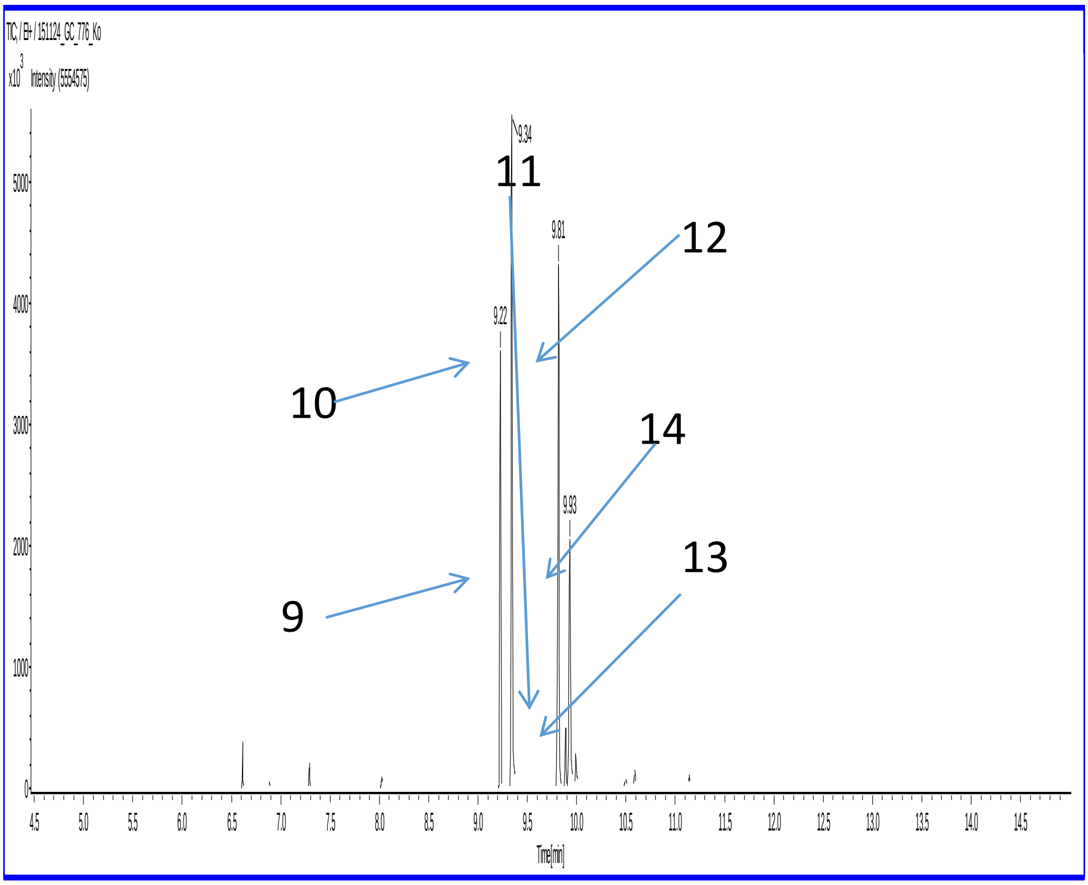

Fig. 11S: GC-MS spectrum of mixture of fatty acids

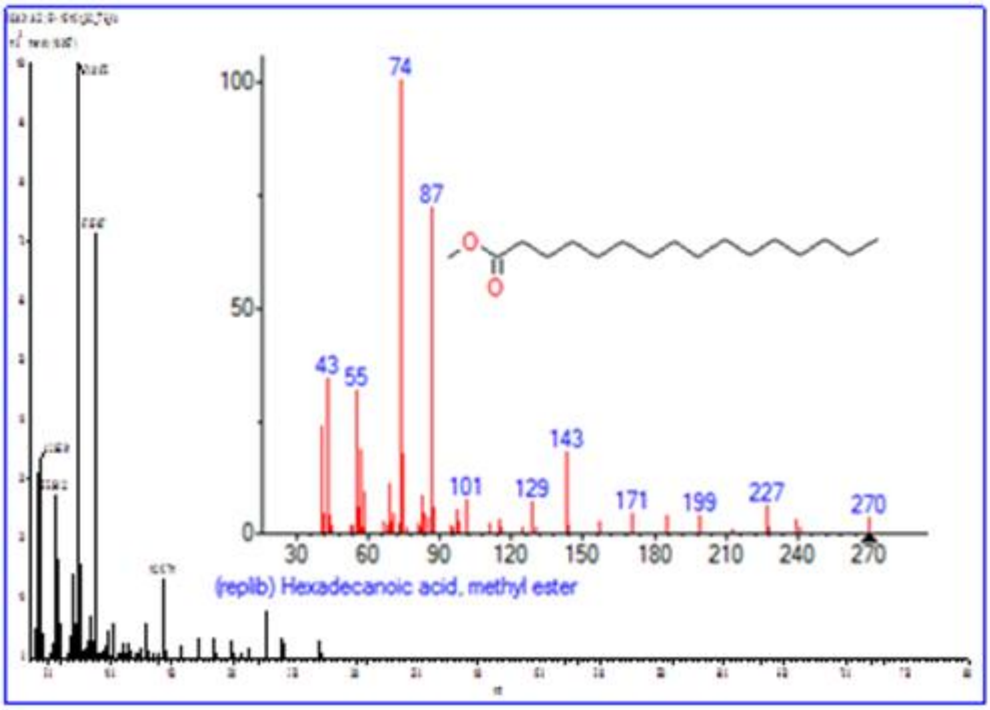

Fig. 12S: identification of compound 9

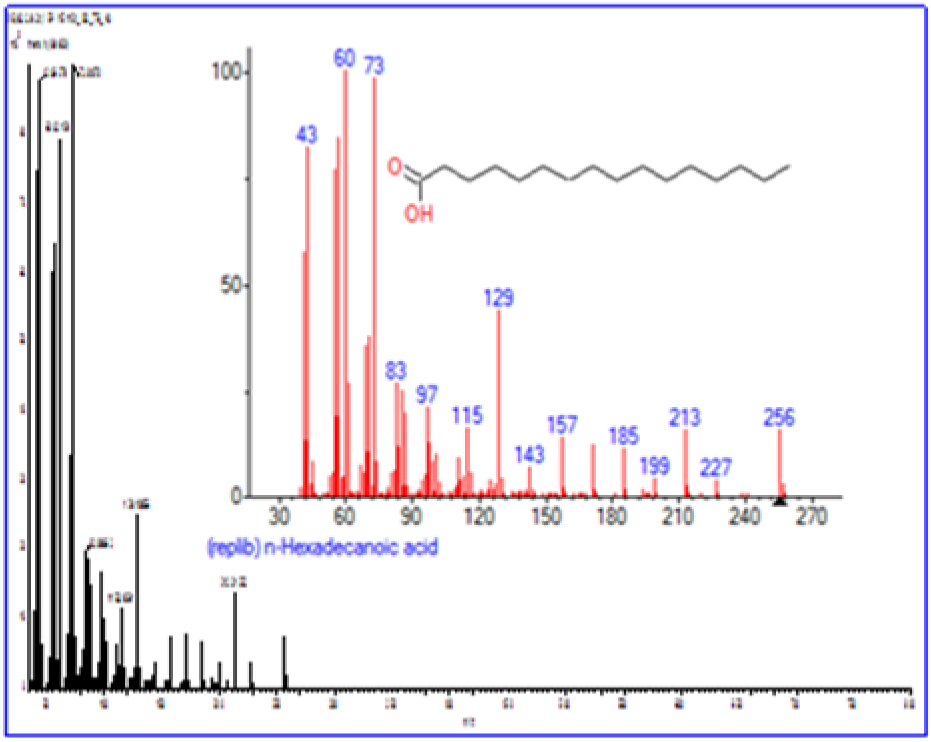

Fig. 13S: identification of compound 10

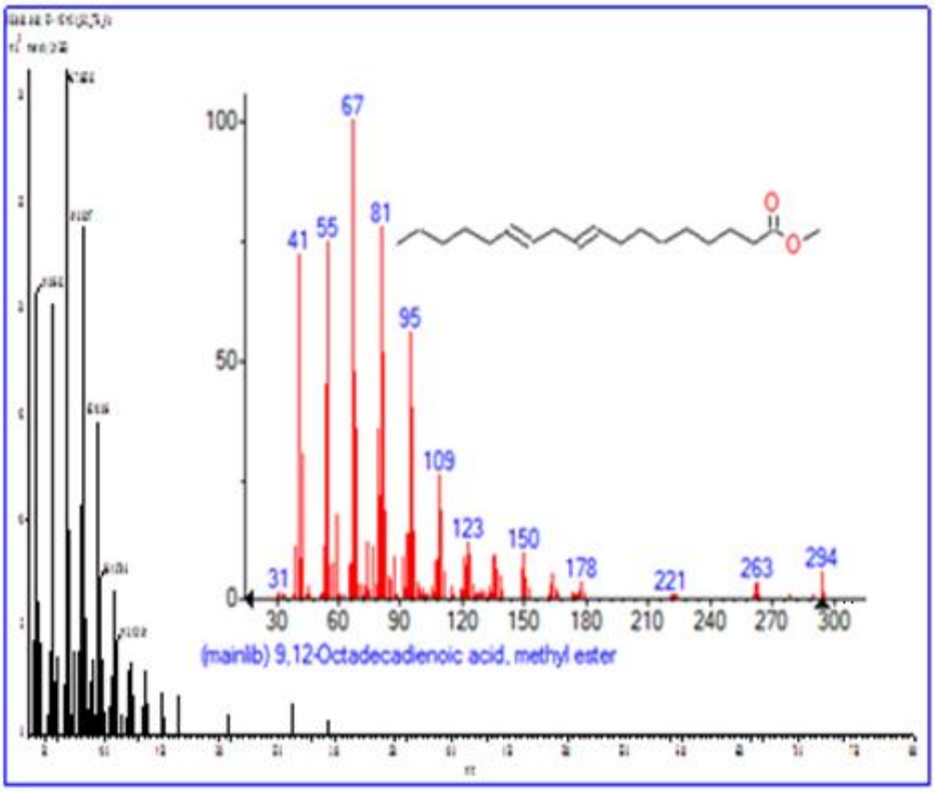

Fig. 14S: identification of compound 11

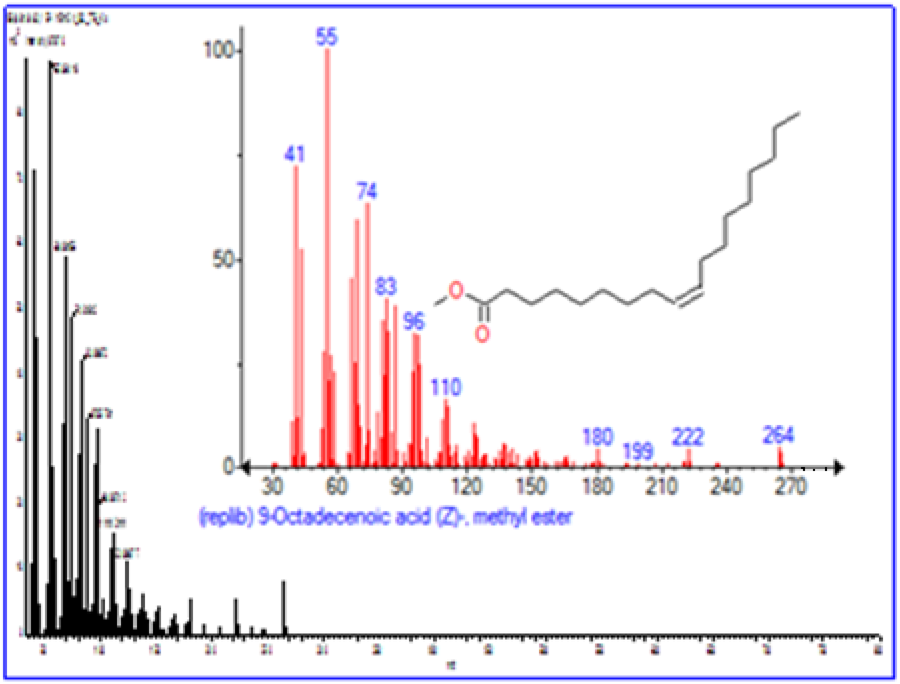

Fig. 15S: identification of compound 12

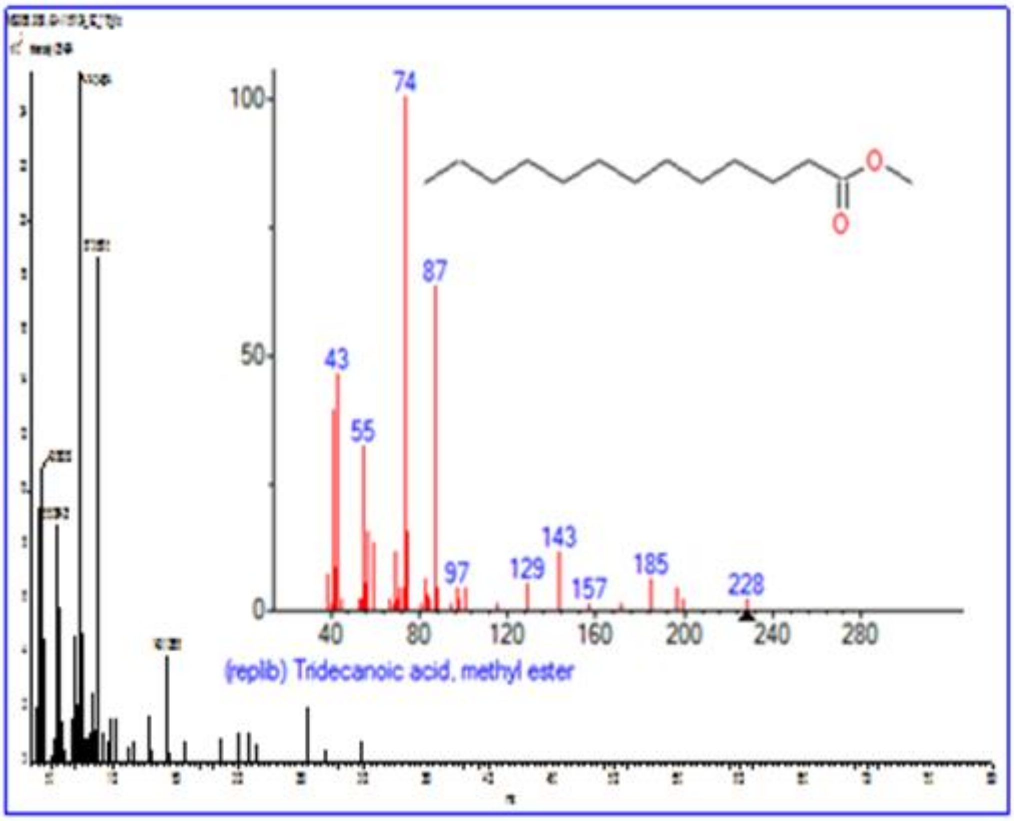

Fig. 16S: identification of compound 13

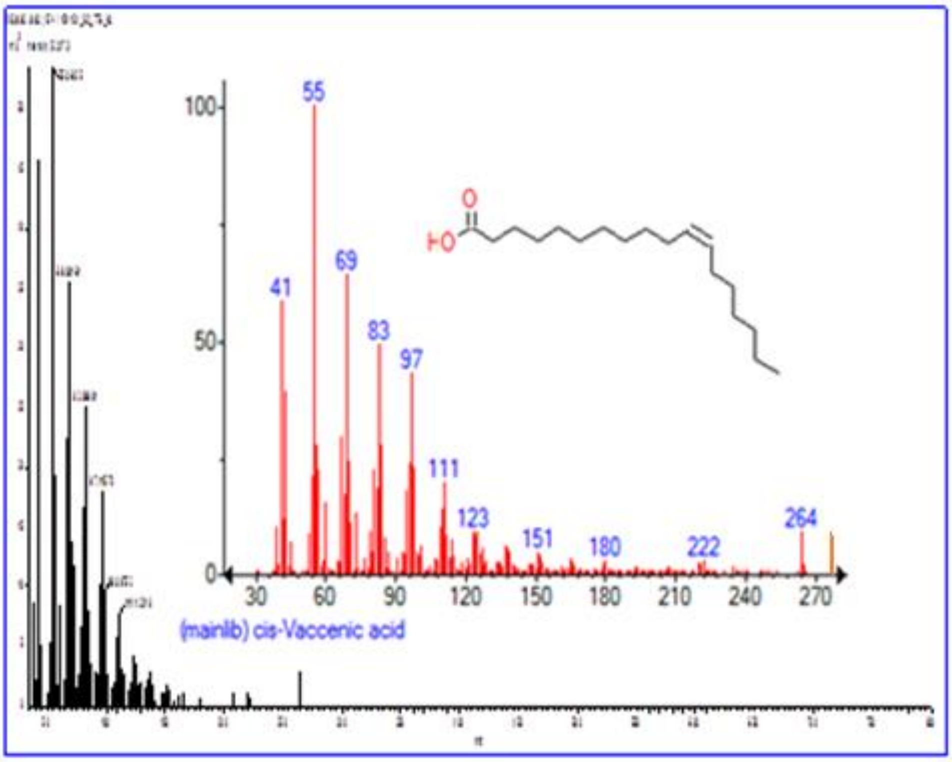

Fig. 17S: identification of compound 14

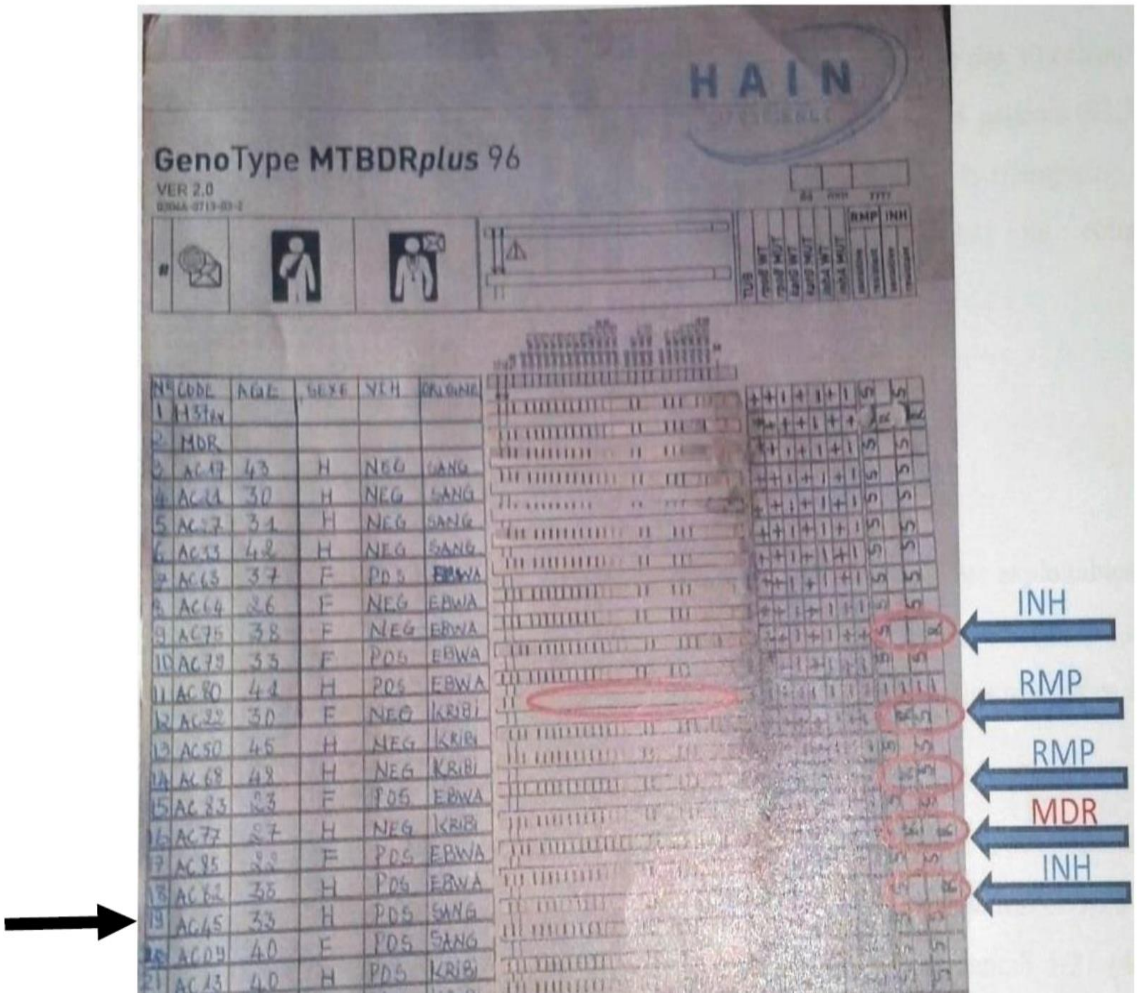

Figure 18S. Genotype profile of *M. tuberculosis* codified AC 45

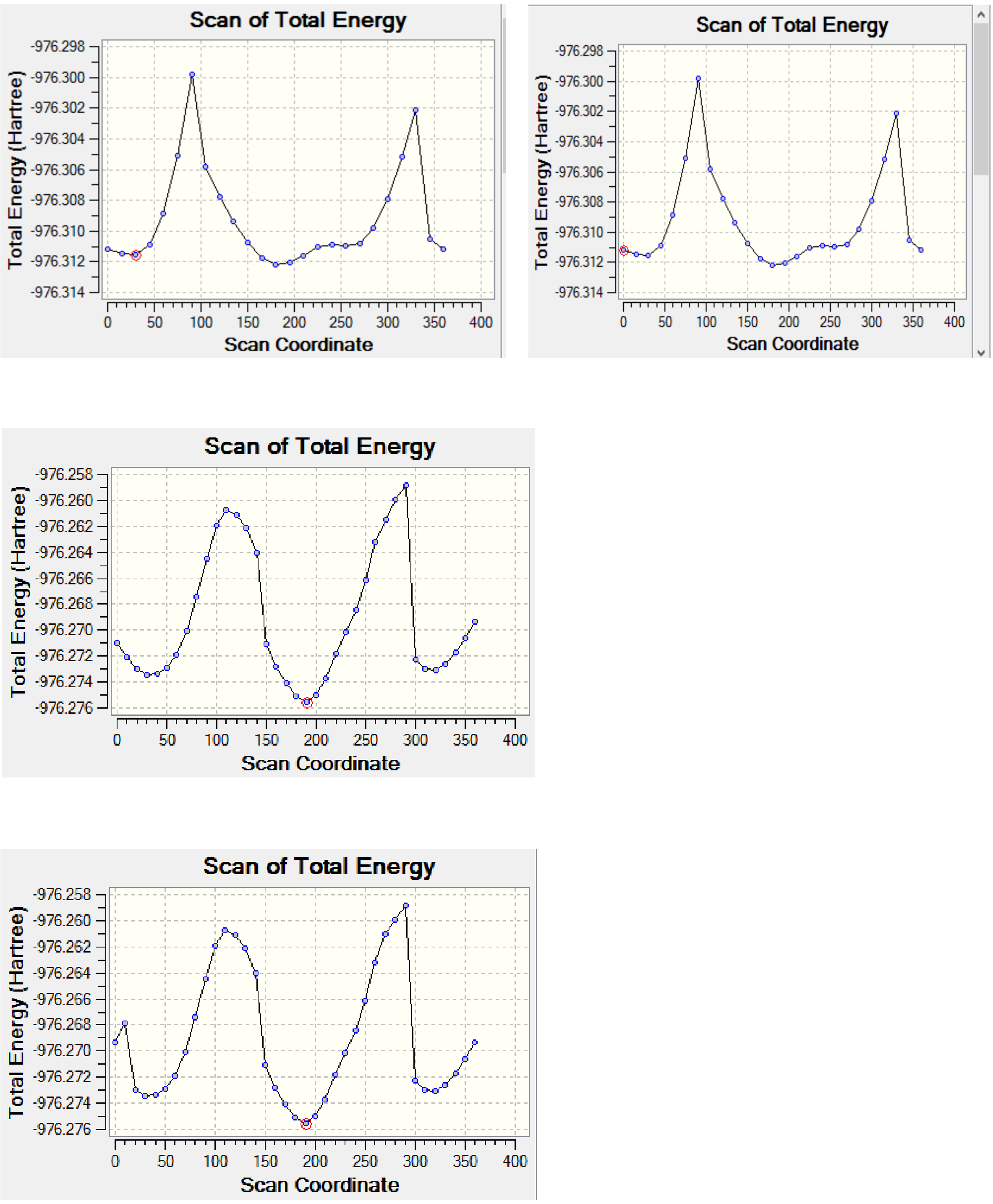

Fig. 18S. Conformational analyses of compound **2**.at B3LYP/6–31G(d).

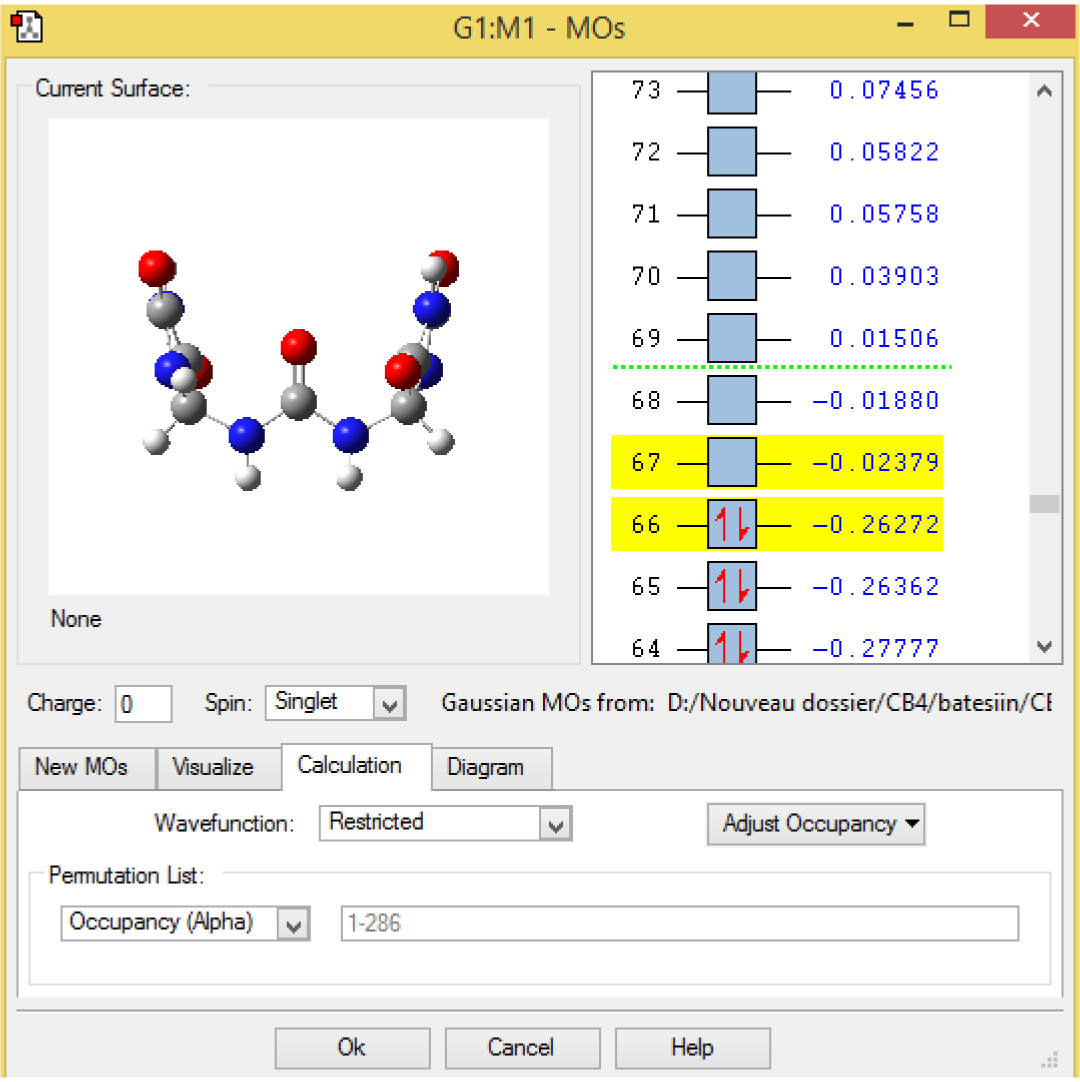

Fig. 19**S**. Graph representing *HOMO*/*LUMO* at B3LYP/6–31G(d) of **2**.

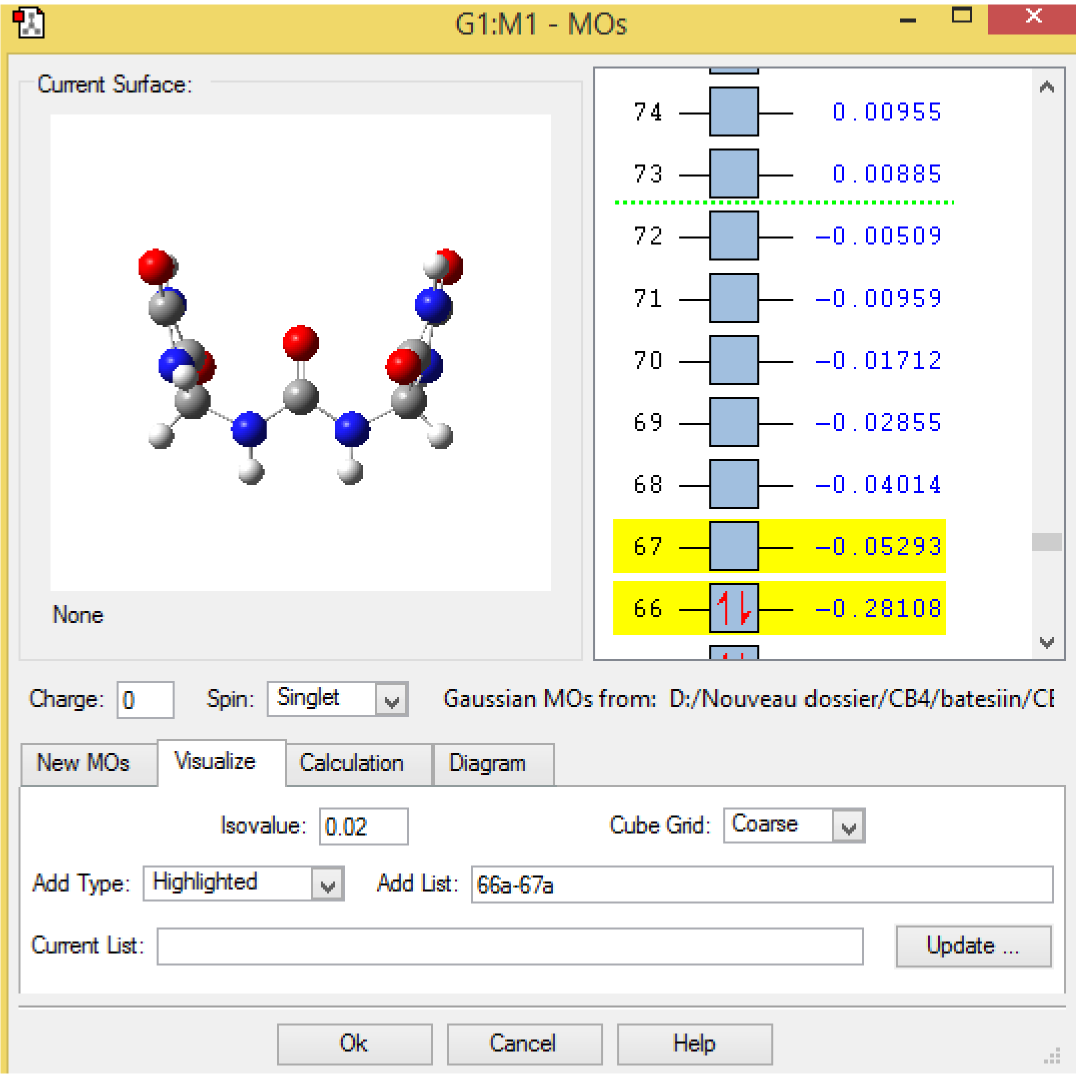

Fig. 20S. Graph representing *HOMO*/*LUMO* at B3LYP/6–311++G(d,p) of **2**.

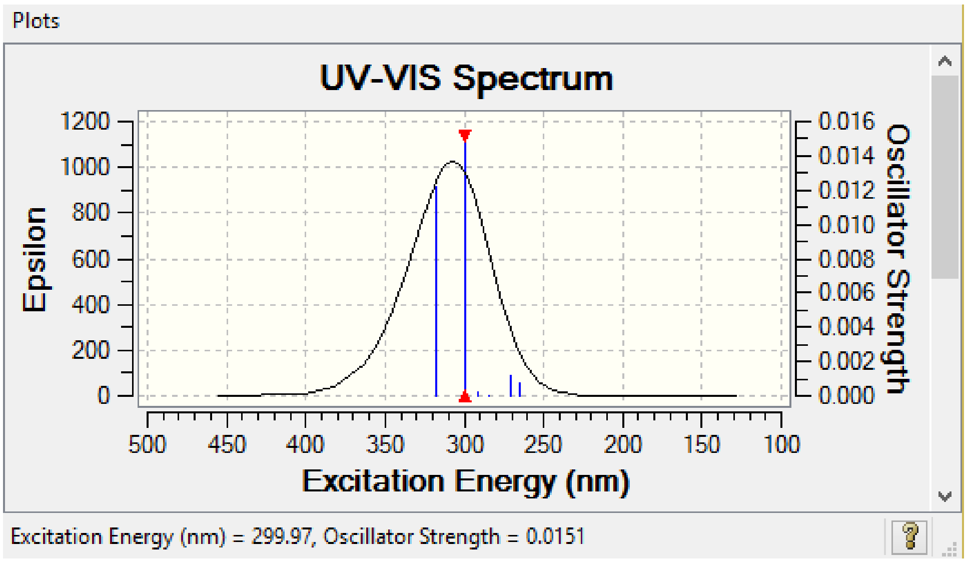

Fig. 21S. UV–visible spectrum of **2** in chloroform at B3LYP/6–31+G(d,p).

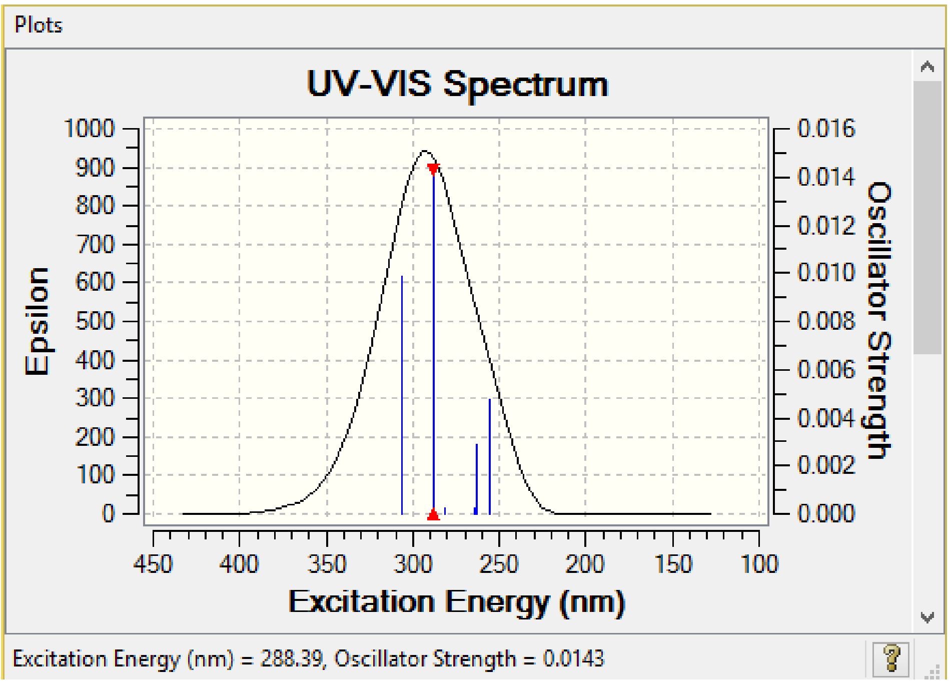

Fig. 22**S**. UV–visible spectrum of **2** in ethanol at B3LYP/6–31+G(d,p).

Table 1S. Cartesian coordinates for the optimized geometry of **2** at B3LYP/6–31G(d).

1	6	0	1.990032	-2.111672	1.461033
2	6	0	0.219987	-0.475880	0.996033
3	6	0	-1.411621	1.279274	0.463481
4	8	0	-0.656484	-1.252445	1.344293
5	7	0	1.573169	-0.764838	1.111606
6	7	0	-0.051146	0.771130	0.449476
7	1	0	3.060042	-2.165672	1.227434
8	1	0	0.669692	1.477011	0.525298
9	1	0	-1.335375	2.350190	0.240518
10	1	0	2.205226	-0.270342	0.495388
11	6	0	1.259527	-3.234339	0.664293
12	7	0	1.724659	-2.493880	2.830412
13	6	0	0.880191	-3.582644	2.930257
14	7	0	0.746416	-4.082070	1.615550
15	6	0	-2.141040	1.116603	1.831166
16	6	0	-3.380998	0.010259	0.206864
17	7	0	-2.319343	0.610420	-0.442341
18	7	0	-3.314187	0.462453	1.543374
19	8	0	0.382588	-4.054169	3.925972
20	8	0	1.207301	-3.324378	-0.540976
21	8	0	-1.741545	1.525840	2.897144
22	8	0	-4.227163	-0.717238	-0.258126
23	1	0	-3.941781	0.102106	2.249883
24	1	0	0.109043	-4.838814	1.405767
25	1	0	1.770274	-1.836519	3.596177
26	1	0	-2.023878	0.248414	-1.338041

C	6	0	-2.452620	-1.105897	-0.077543
C	6	0	-0.000144	-1.009672	-0.042834
C	6	0	2.452754	-1.106983	-0.030894
O	8	0	-0.000312	0.210437	0.019413
N	7	0	-1.162420	-1.767602	0.008279
N	7	0	1.162107	-1.757845	-0.171986
H	1	0	-3.182603	-1.896121	-0.289857
H	1	0	1.131580	-2.719294	0.141556
H	1	0	3.184099	-1.913550	0.099384
H	1	0	-1.129866	-2.691253	-0.403492
C	6	0	-2.549114	-0.036397	-1.207000
N	7	0	-2.822242	-0.340896	1.092866
C	6	0	-3.006699	1.001026	0.821343
N	7	0	-2.978398	1.111575	-0.586761
C	6	0	2.556535	-0.151669	1.196169
C	6	0	3.000464	1.077670	-0.725160
N	7	0	2.814655	-0.231938	-1.124129
N	7	0	2.981496	1.051305	0.686983
O	8	0	-3.197311	1.902877	1.603472
O	8	0	-2.318928	-0.225750	-2.379462
O	8	0	2.334615	-0.453562	2.346383
O	8	0	3.185703	2.051174	-1.417573
H	1	0	3.018985	1.905607	1.227023
H	1	0	-3.012340	2.014391	-1.041387
H	1	0	-2.564144	-0.621179	2.028508
H	1	0	2.550725	-0.420669	-2.080866

E(RB3LYP/6–31G(d)) = – 976.275521828

Table 2S. Cartesian coordinates for the optimized geometry of **2** at B3LYP/6–311++ G(d,p).

1	6	0	-2.457049	-1.100518	-0.083942
2	6	0	0.000071	-0.997847	-0.048472
3	6	0	2.457247	-1.102141	-0.022962
4	8	0	-0.000254	0.216482	0.009918
5	7	0	-1.163403	-1.750538	-0.013955
6	7	0	1.163943	-1.743132	-0.155011
7	1	0	-3.182563	-1.889978	-0.302045
8	1	0	1.130692	-2.715937	0.109743
9	1	0	3.183184	-1.908498	0.118418
10	1	0	-1.129641	-2.693422	-0.370857
11	6	0	-2.576862	-0.022569	-1.202107
12	7	0	-2.830564	-0.347087	1.093948
13	6	0	-3.063133	0.984237	0.832816
14	7	0	-3.016131	1.112331	-0.572034
15	6	0	2.576523	-0.136367	1.193458
16	6	0	3.062200	1.061266	-0.735486
17	7	0	2.830333	-0.238992	-1.123135
18	7	0	3.015175	1.053974	0.675172
19	8	0	-3.296389	1.869587	1.615296
20	8	0	-2.355109	-0.198357	-2.373054
21	8	0	2.354897	-0.423789	2.342136
22	8	0	3.294955	2.017722	-1.429419
23	1	0	3.085384	1.908287	1.208998
24	1	0	-3.086805	2.013878	-1.021444
25	1	0	-2.623077	-0.646175	2.033898
26	1	0	2.622951	-0.446633	-2.087443

C	6	0	-2.456975	-1.104095	-0.042236
C	6	0	-0.000001	-1.000493	-0.000005
C	6	0	2.456974	-1.104096	0.042212
O	8	0	-0.000003	0.215239	0.000016
N	7	0	-1.163998	-1.750349	0.065053
N	7	0	1.163996	-1.750343	-0.065085
H	1	0	-3.181834	-1.902923	-0.225647
H	1	0	1.129004	-2.709306	0.245906
H	1	0	3.181832	-1.902928	0.225605
H	1	0	-1.129006	-2.709291	-0.246006
C	6	0	-2.570936	-0.081041	-1.211422
N	7	0	-2.835714	-0.294882	1.096328
C	6	0	-3.066380	1.022432	0.770475
N	7	0	-3.012634	1.082917	-0.638665
C	6	0	2.570942	-0.081066	1.211419
C	6	0	3.066379	1.022447	-0.770457
N	7	0	2.835715	-0.294861	-1.096338
N	7	0	3.012643	1.082902	0.638684
O	8	0	-3.302916	1.944390	1.508425
O	8	0	-2.343703	-0.312903	-2.371507
O	8	0	2.343713	-0.312949	2.371500
O	8	0	3.302905	1.944422	-1.508389
H	1	0	3.080734	1.961841	1.131209
H	1	0	-3.080724	1.961866	-1.131171
H	1	0	-2.632849	-0.548553	2.050533
H	1	0	2.632818	-0.548504	-2.050544

E(RB3LYP) = – 976.586390118

Table 3S. Shielding tensors (σ) at B3LYP/6-31g(d,p) and experimental chemical shifts (δ_{exp}) of carbons of compound **2**.

**Position**	σ (ppm)	δ_{exp} (ppm)	δ_{calc} (ppm)
C(2)	44.7895	156.8	156.1
C(4)	28.2611	173.6	174.4
C(5)	126.3013	62.4	61.7
C(7)	45.0912	157.4	157.0

Table 4S. Shielding tensors (σ) at MPW1PW91/6–31+G(d,p) and experimental chemical shifts (δ_{exp}) of carbons of compound **2**.

**Position**	σ (ppm)	δ_{exp} (ppm)	δ_{calc} (ppm)
C(2)	44.9493	156.8	157.0
C(4)	28.4488	173.6	174.6
C(5)	129.3335	62.4	62.7
C(7)	45.5375	157.4	158.0

Table 5S. Electronic and thermodynamic parameters of **2** at B3LYP/6–31G(d).

	E (Thermal)	C_V	S

	kcal.mol^{-1}	cal.mol^{-1}.K^{-1}	cal.mol^{-1}.K^{-1}
Total	127.176	58.872	129.784
Electronic	0.000	0.000	0.000
Translational	0.889	2.981	42.521
Rotational	0.889	2.981	33.158
Vibrational	125.399	52.910	54.105
Vibration 1	0.594	1.984	5.765
Vibration 2	0.594	1.982	5.506
Vibration 3	0.595	1.979	4.985
Vibration 4	0.600	1.963	3.888
Vibration 5	0.600	1.962	3.854
Vibration 6	0.608	1.935	3.149
Vibration 7	0.620	1.896	2.595
Vibration 8	0.621	1.892	2.557
Vibration 9	0.624	1.885	2.484
Vibration 10	0.646	1.815	1.984
Vibration 11	0.662	1.764	1.734
Vibration 12	0.685	1.697	1.488
Vibration 13	0.710	1.623	1.279
Vibration 14	0.758	1.491	0.998
Vibration 15	0.758	1.490	0.998
Vibration 16	0.768	1.465	0.953
Vibration 17	0.770	1.459	0.944
Vibration 18	0.788	1.414	0.873
Vibration 19	0.811	1.356	0.789
Vibration 20	0.816	1.343	0.772
Vibration 21	0.884	1.185	0.588
Vibration 22	0.911	1.128	0.533
Vibration 23	0.940	1.068	0.479
Vibration 24	0.942	1.064	0.477

Table 6S. Electronic and thermodynamic parameters of **2** at B3LYP/6–311++G(d,p).

		E (Thermal) Kcal.mol^{-1}	C_V cal.mol^{-1}.K^{-1}	S cal.mol^{-1}.K^{-1}
Total		126.495	59.237	130.565
Electronic		0.000	0.000	0.000
Translational		0.889	2.981	42.521
Rotational		0.889	2.981	33.191
Vibrational		124.718	53.275	54.853
Vibration	1	0.594	1.984	5.773
Vibration	2	0.594	1.982	5.521
Vibration	3	0.595	1.980	5.089
Vibration	4	0.600	1.964	3.929
Vibration	5	0.600	1.963	3.916
Vibration	6	0.608	1.935	3.146
Vibration	7	0.620	1.895	2.591
Vibration	8	0.621	1.895	2.583
Vibration	9	0.622	1.890	2.541
Vibration	10	0.643	1.823	2.026
Vibration	11	0.661	1.767	1.750
Vibration	12	0.685	1.694	1.481
Vibration	13	0.708	1.630	1.296
Vibration	14	0.736	1.550	1.113
Vibration	15	0.747	1.522	1.056
Vibration	16	0.759	1.489	0.995
Vibration	17	0.764	1.475	0.971
Vibration	18	0.773	1.451	0.930
Vibration	19	0.806	1.369	0.806
Vibration	20	0.814	1.349	0.780
Vibration	21	0.882	1.191	0.595
Vibration	22	0.906	1.138	0.542
Vibration	23	0.938	1.071	0.482
Vibration	24	0.942	1.063	0.476

Table 7S. Excitation energies and oscillator strengths of **2** in $CHCl_3$ at B3LYP/6–31+G(d,p).

Excited State 1: Singlet-A 3.8954 eV 318.29 nm f = 0.0122 <S**2>=0.000

H–3 → L 0.14458

H–2 → L 0.18828

H–1 → L 0.63487

This state for optimization and/or second-order correction.

Total Energy, E(TD-HF/TD-KS) = -976.021677358

Copying the excited state density for this state as the 1-particle RhoCI density.

Excited State 2: Singlet-A 4.1332 eV 299.97 nm f=0.0151 <S**2>=0.000

H–5 → L -0.12204

H–3 → L 0.26295

H–2 → L 0.55559

H–1 → L -0.22816

H–1 → L+2 -0.17440

Excited State 3: Singlet-A 4.2614 eV 290.95 nm f=0.0002 <S**2>=0.000

H → L 0.15406

H → L 0.67051

H → L 0.10053

Excited State 4: Singlet-A 4.3524 eV 284.86 nm f=0.0000 <S**2>=0.000

H → L 0.68775

H → L -0.14940

Excited State 5: Singlet-A 4.5856 eV 270.38 nm f=0.0012 <S**2>=0.000

H–5 → L 0.50163

H–5 → L+2 0.11853

H–4 → L -0.19248

H–2 → L 0.17979

H–1 → L+2 0.34446

Excited State 6: Singlet-A 4.6727 eV 265.34 nm f=0.0007 <S**2>=0.000

H–6 → L -0.12195

H–5 → L -0.10366

H–4 → L 0.30015

H–3 → L 0.48758

H–2 → L -0.25917

H–1 → L -0.10994

H–1 → L+2 0.21890

Table 8S. Excitation energies and oscillator strengths of **2** in EtOH at B3LYP/6–31+G(d,p).

Excited State 1: Singlet-A 4.0514 eV 306.03 nm f=0.0099 <S**2>=0.000

H–8 → L -0.11109

H–3 → L 0.10020

H–2 → L -0.18726

H → L 0.63954

This state for optimization and/or second-order correction.

Total Energy, E(TD-HF/TD-KS) = -976.030682697

Copying the excited state density for this state as the 1-particle RhoCI density.

Excited State 2: Singlet-A 4.2992 eV 288.39 nm f = 0.0143 <S**2>=0.000

H–4 → L 0.11974

H–3 → L -0.12646

H–2 → L 0.59334

H → L 0.17578

H → L+2 0.22259

Excited State 3: Singlet-A 4.4026 eV 281.62 nm f=0.0002 <S**2>=0.000

H–1 → L+2 0.68009

Excited State 4: Singlet-A 4.6842 eV 264.68 nm f=0.0002 <S**2>=0.000

H–5 → L 0.14469

H–1 → L 0.67282

Excited State 5: Singlet-A 4.7157 eV 262.92 nm f=0.0029 <S**2>=0.000

H–5 → L 0.25665

H–4 → L 0.29579

H–4 → L+2 0.12092

H–3 → L 0.19842

H–2 → L -0.20075

H–2 → L+2 0.14680

H–1 → L -0.12424

H → L -0.14877

H → L+2 0.38483

Excited State 6: Singlet-A 4.8542 eV 255.42 nm f=0.0047 <S**2>=0.000

H–5 → L 0.45500

H–4 → L 0.29489

H–3 → L -0.15182

H–2 → L+2 -0.12704

H–1 → L	-0.10526
H → L	0.12310
H → L+2	-0.33526
